# Native elongating transcript sequencing reveals global anti-correlation between sense and antisense nascent transcription in fission yeast

**DOI:** 10.1261/rna.063446.117

**Published:** 2018-02

**Authors:** Maxime Wery, Camille Gautier, Marc Descrimes, Mayuko Yoda, Hervé Vennin-Rendos, Valérie Migeot, Daniel Gautheret, Damien Hermand, Antonin Morillon

**Affiliations:** 1 ncRNA, epigenetic and genome fluidity, Institut Curie, PSL Research University, CNRS UMR 3244, Université Pierre et Marie Curie, 75248 Paris Cedex 05, France; 2 URPHYM, Namur Research College (NARC), University of Namur, Namur 5000, Belgium; 3 Institute for Integrative Biology of the Cell, CNRS, CEA, Université Paris Sud, 91405, Orsay Cedex, France

**Keywords:** antisense transcription, Exo2, fission yeast, lncRNA, NET-seq

## Abstract

Antisense transcription can regulate sense gene expression. However, previous annotations of antisense transcription units have been based on detection of mature antisense long noncoding (aslnc)RNAs by RNA-seq and/or microarrays, only giving a partial view of the antisense transcription landscape and incomplete molecular bases for antisense-mediated regulation. Here, we used native elongating transcript sequencing to map genome-wide nascent antisense transcription in fission yeast. Strikingly, antisense transcription was detected for most protein-coding genes, correlating with low sense transcription, especially when overlapping the mRNA start site. RNA profiling revealed that the resulting aslncRNAs mainly correspond to cryptic Xrn1/Exo2-sensitive transcripts (XUTs). ChIP-seq analyses showed that antisense (as)XUT's expression is associated with specific histone modification patterns. Finally, we showed that asXUTs are controlled by the histone chaperone Spt6 and respond to meiosis induction, in both cases anti-correlating with levels of the paired-sense mRNAs, supporting physiological significance to antisense-mediated gene attenuation. Our work highlights that antisense transcription is much more extended than anticipated and might constitute an additional nonpromoter determinant of gene regulation complexity.

## INTRODUCTION

Transcriptomics developments have led to the discovery that eukaryotic genomes are pervasively transcribed, i.e., each nucleotide (nt) can be virtually transcribed into RNA (Berretta and Morillon 2009; Clark et al. 2011). This applies not only to intergenic regions but also to the DNA strand antisense to genes.

Antisense transcription is now recognized as an important regulator of gene expression (Pelechano and Steinmetz 2013), directly affecting sense transcription by transcriptional interference (Latos et al. 2012; Nguyen et al. 2014). Alternatively, but not exclusively, it can produce regulatory antisense long noncoding RNA (aslncRNAs) acting on sense gene expression in *cis* or in *trans*, as shown in the budding yeast *Saccharomyces cerevisiae* (Camblong et al. 2007, 2009; Uhler et al. 2007; Berretta et al. 2008; Houseley et al. 2008; Pinskaya et al. 2009; van Werven et al. 2012), in plant (Swiezewski et al. 2009), and in mammalian cells (Lee and Lu 1999; Yap et al. 2010).

Despite their regulatory importance, aslncRNAs have been poorly studied. One reason for the lack of global information on aslncRNAs appears to be their high instability. For instance, in *S. cerevisiae*, we described a class of regulatory aslncRNAs referred to as Xrn1-sensitive unstable transcripts (XUTs), as they are targeted by the 5′–3′ cytoplasmic Xrn1 exoribonuclease (Van Dijk et al. 2011), mainly through the nonsense-mediated decay (NMD) pathway (Wery et al. 2016). Among them, we defined a subgroup for which the sense-paired genes undergo antisense-mediated transcriptional silencing (Van Dijk et al. 2011). Importantly, XUTs can accumulate in wild-type (WT) cells under physiological stress, modulating the RNA decay activity (Van Dijk et al. 2011), and other antisense transcripts were shown to respond to cell differentiation programs such as meiosis (Lardenois et al. 2011). This indicates that RNA surveillance pathways can indeed be considered as regulatory switches of gene expression, probably combined to histone modifications (Camblong et al. 2007; Houseley et al. 2008; Pinskaya et al. 2009; Van Dijk et al. 2011; Kim et al. 2012).

In *S. cerevisiae*, antisense (as)XUTs were shown to form double-stranded (ds)RNA with their paired-sense mRNA (Wery et al. 2016). Whether dsRNA formation contributes to asXUT-mediated regulation of gene expression remains unknown. However, this might be a singular feature of *S. cerevisiae*, which has lost the RNA interference (RNAi) system and lacks the RNase III endonuclease Dicer that can process dsRNA structures into small interfering (si)RNAs (Drinnenberg et al. 2009).

To get insights into the mechanisms by which regulatory aslncRNAs affect gene expression and other cellular functions, it is important to determine whether they are conserved in organisms endowed with RNAi and if their regulatory activity is maintained throughout evolution. In this regard, the fission yeast *Schizosaccharomyces pombe* constitutes an attractive model, as it shares RNAi with higher eukaryotes (Volpe et al. 2002).

Previous transcriptomics analyses in *S. pombe* have identified hundreds of aslncRNAs, thereby providing several important and pioneer characterizations of antisense transcription in this organism (Dutrow et al. 2008; Wilhelm et al. 2008; Ni et al. 2010; Rhind et al. 2011; DeGennaro et al. 2013; Eser et al. 2016). Some of these aslncRNAs respond to sexual differentiation (Bitton et al. 2011) or to environmental stress (Leong et al. 2014). However, all these studies were based on mature RNA detection in WT cells, certainly leading to an underestimation of the extent and precise definition of the antisense transcription landscape. Indeed, transcription generally elongates to DNA sequences that are not retrieved in the mature RNA molecule, such as those beyond the polyadenylation site. In addition, antisense transcripts can be cryptic due to their rapid degradation by RNA decay factors and are therefore not detectable in a WT context. Thus, to get a precise view of the antisense transcription landscape, it is necessary to measure transcription per se rather than steady-state levels of mature transcripts.

Different techniques have been recently developed in yeasts to analyze transcription genome-wide, in a strand-specific manner. Some of them, such as four-thiouracil sequencing (Eser et al. 2016) and global/precision run-on sequencing (Booth et al. 2016), use metabolic labeling of nascent RNA, followed by sequencing of the labeled transcripts. However, although the labeling times are short, the labeled transcripts remain sensitive to post-transcriptional degradation. Consequently, their levels do not strictly reflect the synthesis rate. In contrast, native elongating transcript (NET) sequencing is based on purification of elongating RNAPII complex purification, followed by deep sequencing of nascent RNAs’ 3′ extremity (Churchman and Weissman 2011). This RNAPII purification step ensures that only elongating transcripts are captured. To date, NET-seq constitutes the state of the art technique to measure quantitatively and qualitatively the transcription process, in a strand-specific manner, at the nucleotide resolution.

Here, using NET-seq, we determine the landscape of antisense transcription in *S. pombe* and investigate how it affects sense gene expression. We detect antisense transcription for more than half of protein-coding genes. Notably, genes with antisense transcription are less transcribed compared to those without antisense transcription, and this correlates with the overlap by the antisense signal, especially when reaching the sense transcription start site (TSS). To get insights into the nature of the antisense transcripts, we define the XUT aslncRNAs landscape using RNA-seq profiling in cells devoid of the 5′–3′ cytoplasmic RNA decay pathway, and we show that they escape RNAi in fission yeast. We observe that genes with asXUTs display specific chromatin marks. Finally, subsets of asXUTs are up-regulated in cells inactivated for the conserved histone chaperone Spt6, and in diploid cells undergoing meiosis. In both conditions, asXUT accumulation correlates with paired-sense mRNA down-regulation.

Our work indicates that antisense transcription in fission yeast is much more extended than anticipated and might constitute a nonpromoter determinant for gene regulation complexity.

## RESULTS

### Extensive antisense transcription in fission yeast

**FIGURE 1.**
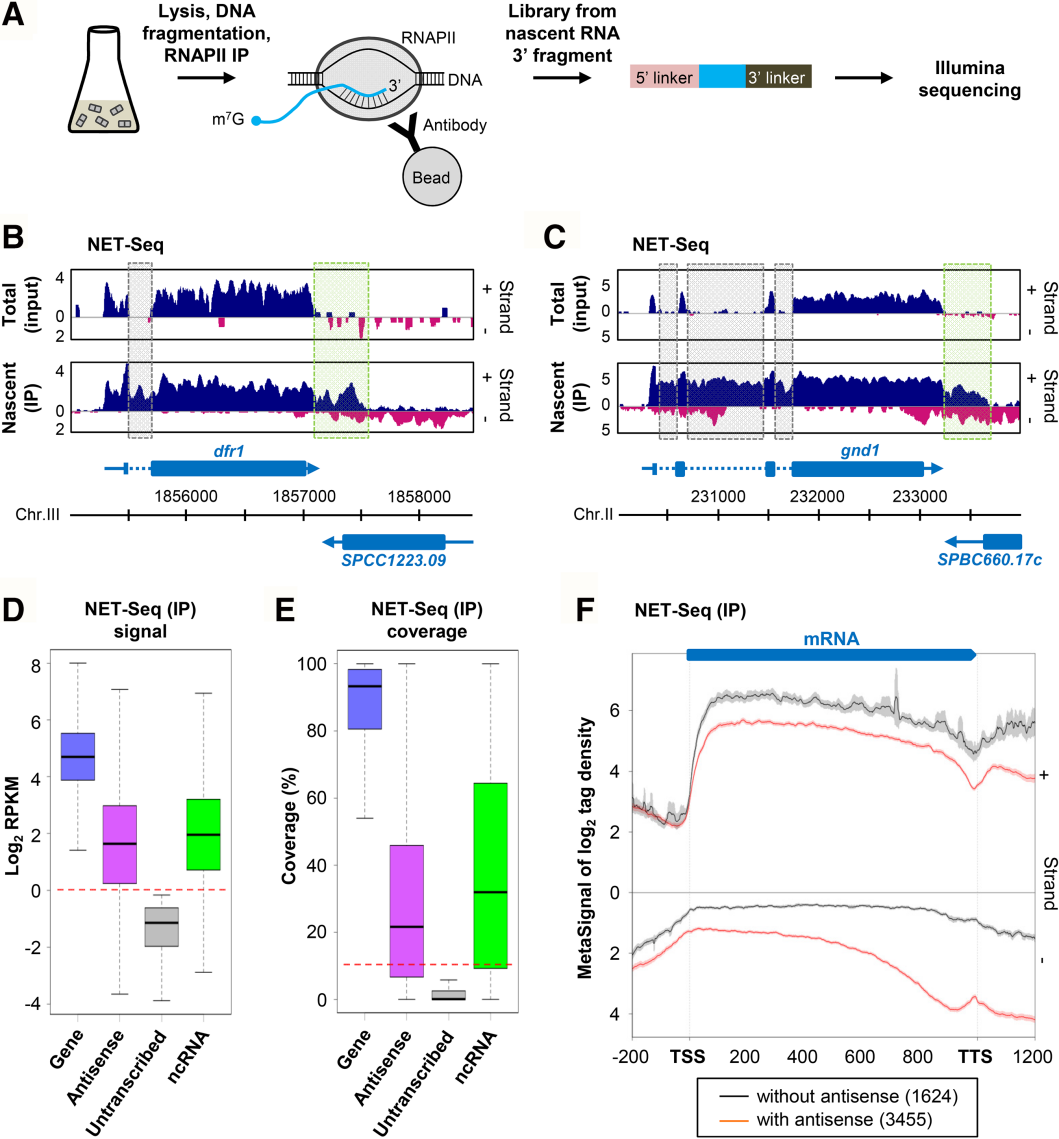
Extensive antisense transcription in fission yeast. (*A*) Overview of NET-seq. Biological duplicates of WT (YAM2492) cells were grown to mid-log phase in rich (YES) medium. After cells’ lysis and DNA fragmentation, ternary RNAPII/DNA/RNA complexes were purified. Strand-specific libraries were then constructed from nascent RNAs 3′ fragments. (*B*,*C*) Snapshots of total RNA (input) and nascent transcript (IP) signals along the *dfr1* (*B*) and *gnd1* (*C*) genes in WT cells. In each panel, the signal corresponding to the + and − strand is shown in blue and pink, respectively. Blue arrow, dashed line, and box represent the mRNA, intron, and coding sequences, respectively. Introns and the region downstream from the polyadenylation site of *dfr1* and *gnd1* are highlighted using dashed gray and green boxes, respectively. The snapshot was produced using VING (Descrimes et al. 2015). (*D*) Box-plot of nascent transcript signal (RPKM, log_2_) in WT cells for transcribed (RPKM >1, blue) or untranscribed (RPKM <1, gray) protein-coding genes, for their antisense (magenta) and for annotated ncRNAs (green). This set corresponds to the 1522 ncRNAs from the current version of Pombase, plus 487 novel ncTU (Eser et al. 2016). The red dashed line indicates the threshold (RPKM >1). (*E*) Box-plot of coverage by nascent transcript reads. Coverage corresponds to the percentage of nucleotides of each element covered by ≥1 uniquely mapped reads, in each of the two biological replicates of the WT strain. The red dashed line indicates the threshold (10%). (*F*) Metagene view of genes with (red lines) or without (black dashed lines) antisense transcription. For each group, normalized coverage (tag/nt, log_2_) along sense (+) and antisense (−) strands were piled up in a strand-specific manner, and the average signal for each strand was plotted. TSS and TTS correspond to gene (mRNA) transcription start site and transcription termination site, respectively. Note that the TSS to TTS region was first scaled to 1000 (virtual) nt, and the signal associated to each nucleotide was scaled upon polynomial interpolation, as previously described (Sinturel et al. 2015). The shading surrounding each line denotes the 95% confidence interval.

To determine the comprehensive landscape of antisense transcription in *S. pombe*, we constructed NET-seq libraries from two biological replicates of WT cells, following immunoprecipitation of RNAPII (Fig. 1A; see also Supplemental Fig. S1A). As a validation that NET-seq detected nascent transcripts, we observed signals from introns and regions beyond the polyadenylation site, which are absent from mature mRNAs (Fig. 1B,C; see also Supplemental Fig. S1B–D). Moreover, nascent transcription signals for mRNAs and annotated ncRNAs showed high reproducibility between replicates (Supplemental Fig. S1E,F).

To identify genes with antisense transcription, we used (i) the nascent transcription signal (RPKM) and (ii) the coverage (proportion of nucleotides covered by ≥1 nascent transcript read in each of the two biological replicates). As a reference, we used a set of 50 untranscribed protein-coding genes, showing the lowest expression at the nascent transcription level (Fig. 1D) and a very low expression at the total RNA level (Supplemental Fig. S1G). The choice of these genes rather than intergenic regions was motivated by the fact that the transcriptional state of the latter remains largely unknown.

The nascent transcription signal for the “untranscribed” set of genes was comprised between 0.07 and 0.89 RPKM (median = 0.45, Fig. 1D), and coverage was ≤10% (median = 0.08%, Fig. 1E). On this basis, we used RPKM >1 and coverage >10% (more than twice the median and higher than the maximum value for the reference set of genes) as thresholds to consider that a region is transcribed.

The signal for most protein-coding genes and 82% of annotated ncRNAs were above the threshold (median RPKM = 25.6 and 3.7, respectively; Fig. 1D). Strikingly, this was also the case for the antisense of 3948 (78%) genes (median RPKM = 3.03; Fig. 1D). Median coverage for protein-coding genes, annotated ncRNAs and the antisense of genes was 93.3%, 32%, and 21.6%, respectively (Fig. 1E). Combining both parameters (RPKM >1 and coverage >10%), 3455 protein-coding genes (68%) displayed antisense transcription in WT cells of *S. pombe*. Notably, global signal and coverage for the antisense of these 3455 genes are in the same range as for the set of annotated ncRNAs (Supplemental Fig. S1H,I), corresponding to 1522 ncRNAs from the current version of Pombase and 487 novel noncoding transcription units (TU) recently annotated (Eser et al. 2016).

Metagene analysis highlighted several fundamental features of sense and antisense transcription in fission yeast (Fig. 1F): (i) It confirms the recent observation made using precision run-on sequencing (Booth et al. 2016) that transcription in fission yeast extends far beyond the mRNA 3′ end; (ii) it reveals that the genes with antisense transcription globally display a peak of nascent antisense signal in the 3′ region; (iii) these genes are globally less transcribed than those without antisense (*P* < 2.2 × 10^−16^, Wilcoxon rank-sum test), which is consistent with previous observations based on RNA labeling (Eser et al. 2016).

In summary, analysis of nascent transcription using NET-seq provides evidence that up to 68% of protein-coding genes in *S. pombe* display antisense transcription, and these genes are less transcribed than those without antisense transcription. This observation is far from the original assumptions based on RNA-seq and microarray data analysis and considerably extends the antisense transcript(ion) as a general property of the gene structure.

### Sense transcription decreases as the overlap by antisense transcription increases

**FIGURE 2.**
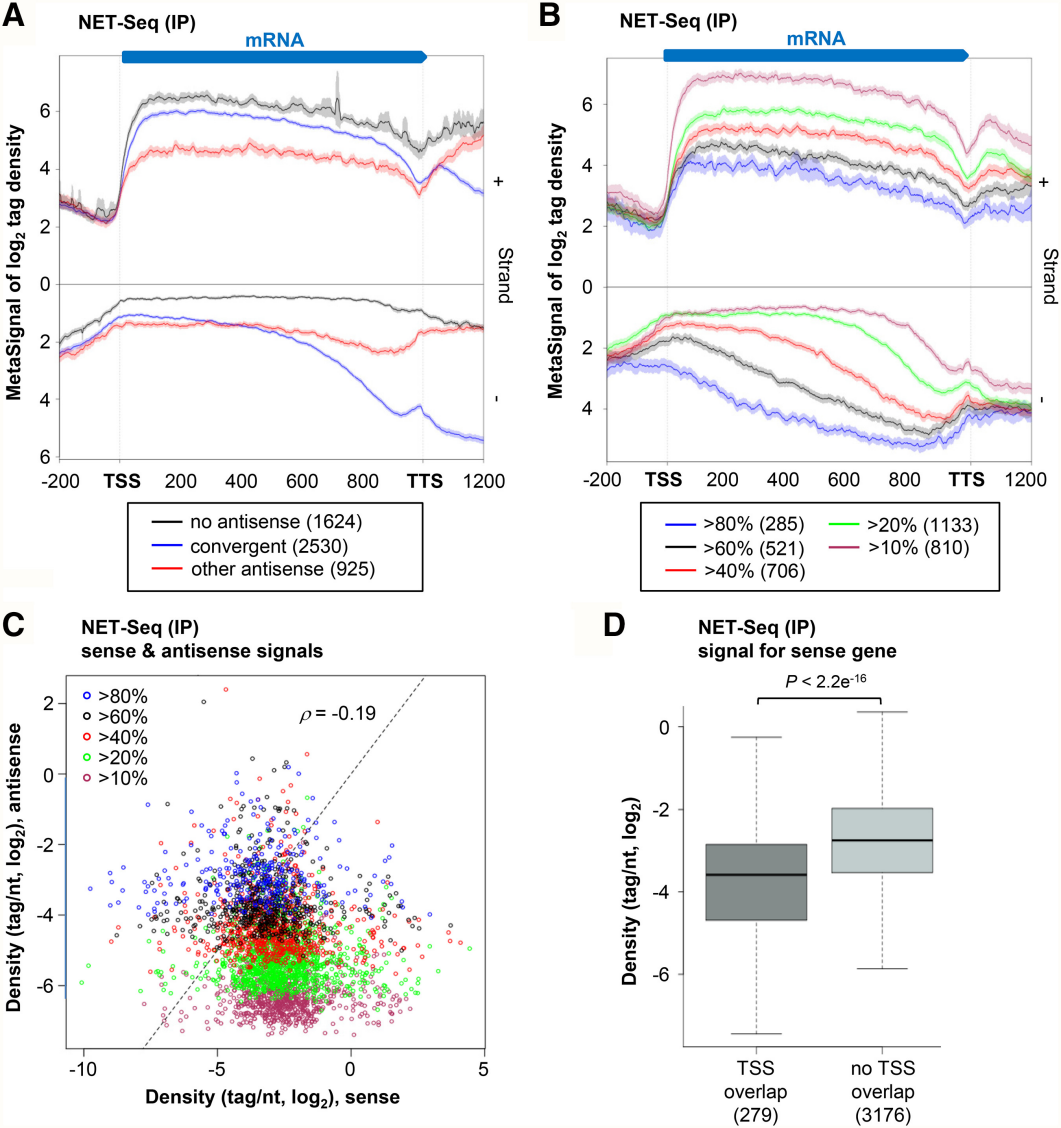
Sense transcription decreases with the overlap by antisense transcription. (*A*) Metagene view of genes without antisense transcription (1624; black), genes with overlapping convergent TU (2530; blue), and genes with antisense but not convergent transcription (925; red). Sense and antisense meta-signals were computed and plotted as described in Figure 1F. (*B*) The 3455 genes with antisense transcription were divided into five classes according to the coverage by antisense signal (see coverage definition in Fig. 1E). For each class, sense and antisense meta-signals were computed and plotted as described above. (*C*) Scatter-plot of sense and antisense nascent transcription signals (tag/nt, log_2_) for the 3455 genes with antisense transcription, divided into five classes as described above. The Spearman's correlation coefficient is indicated. (*D*) The genes with antisense transcription were divided into genes for which it overlaps the TSS (279; dark gray) or not (3176; light gray), according to >80% coverage on the antisense, within a ±100-nt window around the sense TSS. For both classes of genes, NET-seq signal (tag/nt, log_2_ scale) was computed. Data are presented as a box-plot, and the *P*-value obtained upon Wilcoxon rank-sum test is indicated.

The observation of a peak of antisense transcription in the 3′ region of genes raises the hypothesis that this could correspond to overlapping convergent TU. Among the 3455 genes with antisense nascent transcription, 2530 showed overlapping NET-seq signals with a convergent annotated TU (Fig. 2A). This is much more than estimated upon segmentation of RNA-seq signals (Eser et al. 2016), indicating that while convergent TUs largely overlap with each other at the level of nascent transcription, this is globally not the case for the resulting RNAs (see Supplemental Fig. S2A). Illustrative examples of such convergent TUs are shown in Figure 1B,C (see also Supplemental Fig. S1B).

Besides, 925 genes displaying antisense transcription are not convergent to another TU. Metagene analysis revealed a different distribution of the antisense transcription signal for these 925 genes, covering most of the antisense strand (Fig. 2A). Strikingly, these genes were less transcribed than the 2530 convergent genes (Fig. 2A, *P* < 2.2 × 10^−16^, Wilcoxon rank-sum test), suggesting a negative correlation between the level of sense transcription and the extent of the overlap with antisense transcription.

To test this hypothesis, the genes with antisense transcription were separated into five subgroups, according to their coverage by the nascent antisense signal. We then compared the levels of sense transcription for these subgroups. Remarkably, sense transcription significantly decreased as the overlap by the antisense increased (Fig. 2B, see also Supplemental Fig. S2B). Globally, we also noted that levels of sense and antisense transcription are anti-correlated (Spearman correlation coefficient −0.19, Fig. 2C), and for most genes with >80% of overlap by the antisense, the transcription level of the antisense is even stronger than the sense (Fig. 2C; see also Supplemental Fig. S2C).

Finally, overlap of the sense gene TSS appeared to be critical, as genes of which the TSS was overlapped by antisense transcription signal were significantly less transcribed than genes without antisense signal overlapping TSS (*P* < 2.2 × 10^−16^, Wilcoxon rank-sum test; Fig. 2D; see also Supplemental Fig. S2D).

Together, these data reveal a global negative correlation between sense and antisense transcription, and this effect increases with the overlap, in particular over the sense TSS, by antisense transcription.

### Antisense transcripts are mainly cryptic and sensitive to the cytoplasmic 5′–3′ RNA decay

The analysis above revealed that 2530/3455 genes with antisense transcription have convergent overlapping TU. We hypothesized that at least for some of the 925 remaining genes, the antisense signal corresponds to a previously annotated aslncRNA. We retrieved 975/1286 genes with an annotated aslncRNA among the 3455 genes with antisense transcription, and 639 were common to the set of convergent genes (Supplemental Fig. S2E). On the other hand, there were 589 genes for which the origin of the antisense signal detected at the transcriptional level could not be assigned to a convergent TU or an annotated aslncRNA.

Interestingly, at the RNA level, the antisense signal in WT cells for the 925 nonconvergent genes was globally very low, close to the background (Supplemental Fig. S2A), suggesting that the resulting antisense transcripts are unstable. In *S. cerevisiae*, such transcripts are predominantly targeted by the cytoplasmic 5′–3′ exoribonuclease Xrn1, the inactivation of which results in the stabilization of a class of aslncRNAs referred to as XUTs (Van Dijk et al. 2011). Xrn1 is conserved across eukaryotes (Nagarajan et al. 2013), and the orthologous gene in *S. pombe* is *exo2*^+^ (Szankasi and Smith 1996).

**FIGURE 3.**
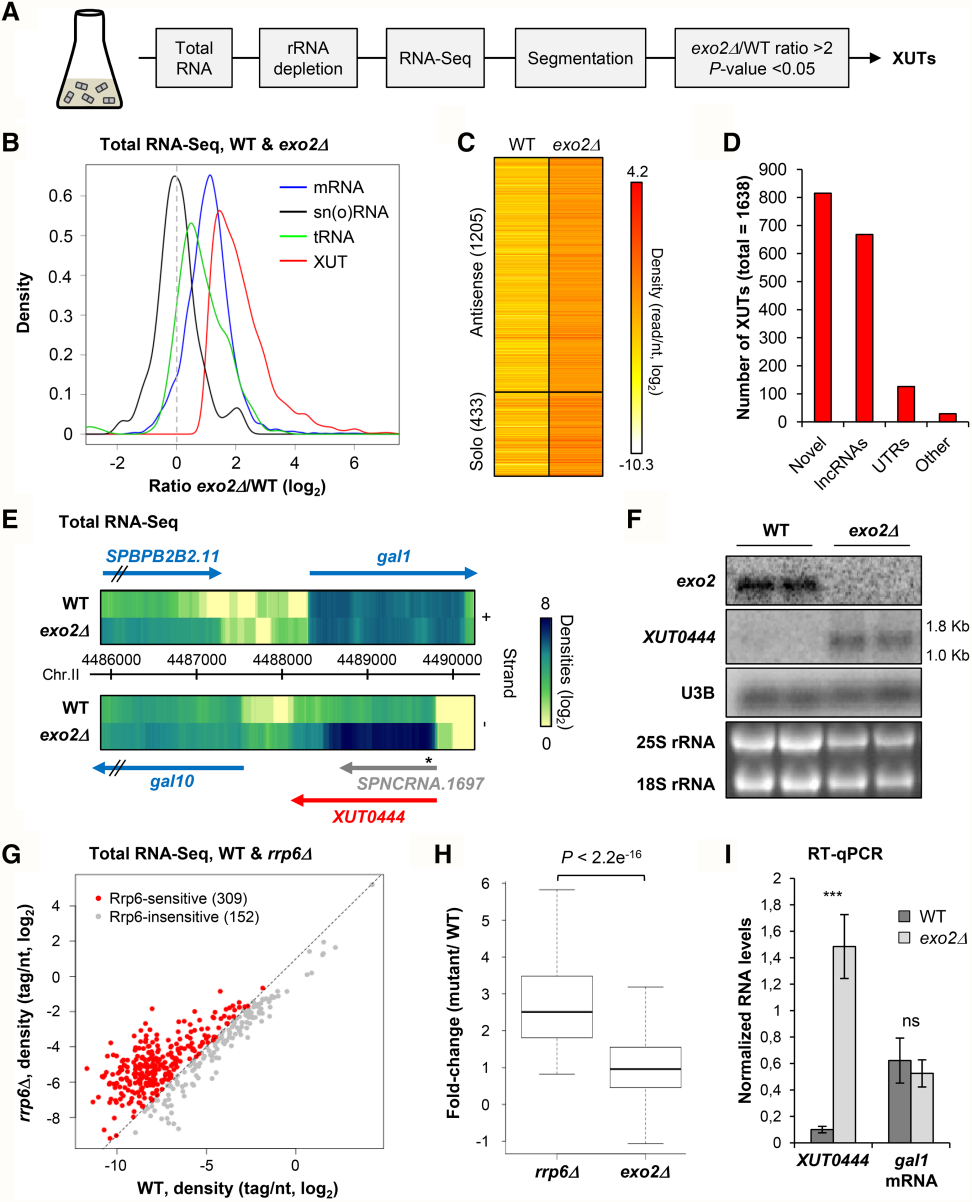
XUT lncRNAs landscape in fission yeast. (*A*) Experimental strategy overview. Total RNA was extracted from exponentially growing YAM2394 (WT) or YAM2397 (*exo2*Δ) cells. After rRNA depletion, strand-specific libraries were constructed and sequenced. Segmentation was performed from uniquely mapped reads. XUTs were identified as segments showing no overlap with ORF, an *exo2*Δ/WT ratio >2, and an adjusted *P* < 0.05 upon differential expression analysis. (*B*) Density plot of *exo2*Δ/WT signal ratio for mRNAs (blue), sn(o)RNAs (black), tRNAs (green), and XUTs (red) upon normalization on sn(o)RNAs. (*C*) Total RNA-seq signal for XUTs in WT and *exo2*Δ are shown as a heatmap. The number of antisense and solo XUTs is indicated. (*D*) Overlap (≥1 nt) between XUTs, previously annotated lncRNAs, UTRs, and other elements (pseudogenes, tRNAs). (*E*) Snapshot of total RNA-seq signal along *gal1* in WT and *exo2*Δ cells. Signal for the + and − strands are visualized as heatmaps in the *upper* and *lower* panels, respectively, using the VING software (Descrimes et al. 2015). The star indicates the position of the probe used to detect *XUT0444* in Fig. 3F. (*F*) Northern blot detection of *XUT0444* (antisense to *gal1*). Transcripts were detected from total RNA extracted from biological duplicates of YAM2394 (WT) and YAM2397 (*exo2*Δ) cells, using ^32^P-labeled oligonucleotides (listed in Supplemental Table S1). Levels of the 18S and 25S rRNAs are visualized from ethidium bromide staining of the gel. Size of the RNA ladder bands is indicated on the *side* of the *XUT0444* panel. *XUT0444* predicted size is 1.7 kb. (*G*) Identification of putative Rrp6-sensitive aslncRNAs. The RNA-seq signal antisense to the 461 genes with antisense transcription but no convergent TU or no annotated aslncRNA/asXUT was computed from previously published total RNA-seq data, obtained from WT and *rrp6*Δ cells (Zhou et al. 2015). Data are presented as a scatter plot of tag densities (log_2_ scale)*,* computed using uniquely mapped reads. The 309 putative Rrp6-sensitive aslncRNAs (*rrp6*Δ/WT ratio >2) are highlighted in red. The black dashed line indicates a twofold increase. (*H*) Box-plot of the *rrp6*Δ/WT and *exo2*Δ/WT ratios for the 309 putative Rrp6-sensitive antisense transcripts. The *P*-value obtained upon Wilcoxon rank-sum test is indicated. (*I*) RT-qPCR analysis of *XUT0444* and *gal1* mRNA levels in WT and *exo2*Δ cells. Strains YAM2400 (WT) and YAM2402 (*exo2*Δ) were grown in rich YES medium to mid-log phase. Levels of *XUT0444* and *gal1* mRNA were determined by strand-specific RT-qPCR from total RNA and normalized on U3B snoRNA level. Data are presented as mean ± standard deviation (SD), calculated from three biological replicates. (***) *P* < 0.001 upon *t*-test; ns, not significant.

To characterize the XUT aslncRNAs’ landscape in fission yeast, we performed strand-specific RNA-seq in WT and *exo2*Δ cells (Fig. 3A), reaching ≥375× genome coverage. Read densities were normalized on sn(o)RNAs that are insensitive to Xrn1/Exo2 (Fig. 3B; Supplemental Fig. S3A), as in *S. cerevisiae* (Van Dijk et al. 2011; Wery et al. 2016). Transcript assembly by the ZINAR segmentation protocol (Wery et al. 2016) identified 1638 XUTs, defined by a greater than twofold enrichment in *exo2*Δ and a significant (*P* < 0.05) differential expression (Fig. 3C; see also Supplemental Fig. S3A). Among them, 815 (49.8%) are novel transcripts; 668 overlap ≥1 nt of previously annotated lncRNAs (Fig. 3D). Figure 3E shows a snapshot of *XUT0444* (antisense to the *gal1* gene), which was detected as a discrete transcript by northern blot (Fig. 3F). Other novel asXUTs were successfully detected by strand-specific RT-qPCR (see Supplemental Fig. 3C–F), reinforcing our genomic profiling approach.

As in *S. cerevisiae*, most XUTs (1205, 73.6%) are antisense to protein-coding genes in *S. pombe* (Fig. 3C). Conversely, 1086 (21.2%) protein-coding genes have asXUTs in fission yeast. GO term finder analysis showed that these genes are significantly enriched for genes involved in nucleic acid metabolism (*P* = 3.60 × 10^−5^), cellular response to DNA damage (*P* = 1.84 × 10^−2^), and chromosome organization (*P* = 3.03 × 10^−2^), but are depleted for genes involved in translation (*P* = 3.46 × 10^−3^).

Unexpectedly, only 128/589 (21.7%) genes for which the antisense signal could not be attributed to a convergent TU or a previously annotated aslncRNA turned out to have asXUT, leaving 461 genes for which the antisense signal origin remains unknown (Supplemental Fig. S3B). However, 309 of them might correspond to genes with antisense cryptic unstable transcripts (CUTs) targeted by the nuclear exosome (Wyers et al. 2005). Indeed, analysis of published total RNA-seq data from cells lacking the Rrp6 subunit of the exosome (Zhou et al. 2015) revealed a greater than twofold increase of the RNA signal antisense to these 309 genes, in the *rrp6*Δ mutant (Fig. 3G,H).

Finally, we asked whether asXUTs’ stabilization in *exo2*Δ correlates with paired-sense mRNAs down-regulation. Strand-specific RT-qPCR analyses on several asXUT/mRNA pairs revealed that this is not the case (Fig. 3I; Supplemental Fig. 3C–F), confirming the RNA-seq profiles (see Fig. 3E; Supplemental Fig. 3G–J). Actually, mRNAs globally accumulate in *exo2*Δ (see Fig. 3B), which is consistent with the role of Xrn1/Exo2 in mRNA turnover, hence hiding the possible regulatory effect of the paired-asXUTs. The question of the effect of asXUT on sense mRNA levels should therefore be addressed in other genetic contexts or physiological conditions for which asXUTs are up-regulated, without inactivating Exo2.

In summary, antisense transcription originates not only from convergent but also from bona fide antisense TU. Among the latter, a majority gives rise to cryptic lncRNAs that are mainly degraded by Exo2 (XUTs), or for a smaller number of cases by Rrp6 (CUTs).

### Antisense XUTs are insulated from RNAi in fission yeast

**FIGURE 4.**
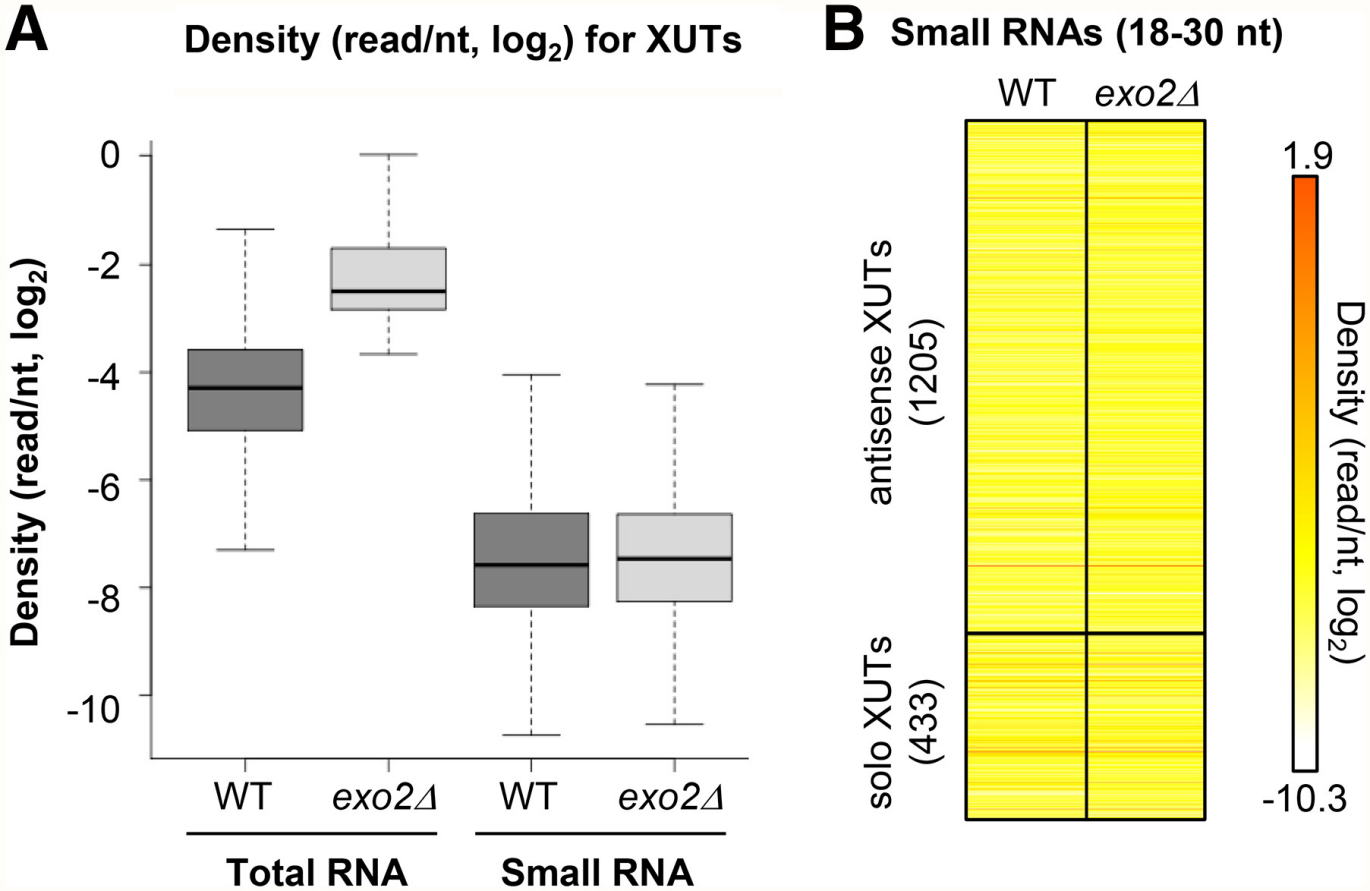
Antisense XUTs are insulated from RNAi in fission yeast. (*A*) Box-plot of tag density (tag/nt, log_2_ scale) for XUTs computed using uniquely mapped reads obtained from sequencing of total or small RNA in YAM2394 (WT) and YAM2397 (*exo2*Δ) strains. (*B*) Heatmap visualization of 18- to 30-nt small RNAs density (tag/nt, log_2_ scale) for antisense and solo XUTs in the WT (YAM2394) and *exo2*Δ (YAM2397) strains.

The observation that the majority of XUTs are also antisense in *S. pombe* raised the question of their interrelation with the RNAi machinery that could in theory generate siRNAs from mRNA/asXUT duplexes, which were shown to exist in *S. cerevisiae* (Wery et al. 2016). Small transcriptome analysis in WT and *exo2*Δ cells filtering 18- to 30-nt small RNAs showed no enrichment of tags in asXUTs (Fig. 4A,B). Similarly, for the mRNAs with asXUTs, we observed no difference of tags densities in the regions overlapped by the asXUTs (i.e., putative dsRNA structure) compared to “solo” (not overlapped) regions in the same mRNAs (Supplemental Fig. S4A). In addition, the reads mapped on these regions did not show the typical size (22–23 nt) expected for genuine siRNAs produced by Dicer in *S. pombe* (Supplemental Fig. S4B,C).

Together, these observations suggest that asXUTs do not form dsRNA or more likely that Dicer and asXUTs are in distinct subcellular compartments. Indeed, XUTs presumably localize into the cytoplasm where they are targeted by Exo2 while Dicer is restricted to the nucleus and associates to specific chromosomal regions, mainly the centromeric repeats (Woolcock and Bühler 2013). Accordingly, we observed that the majority of small RNAs in WT and *exo2*Δ strains are 22- to 23-nt RNAs derived from centromeric repeats (Supplemental Fig. S4D,E).

In conclusion, asXUTs coexist with RNAi but are not targeted by it in fission yeast.

### Expression of asXUTs is associated to specific patterns of histone modifications

**FIGURE 5.**
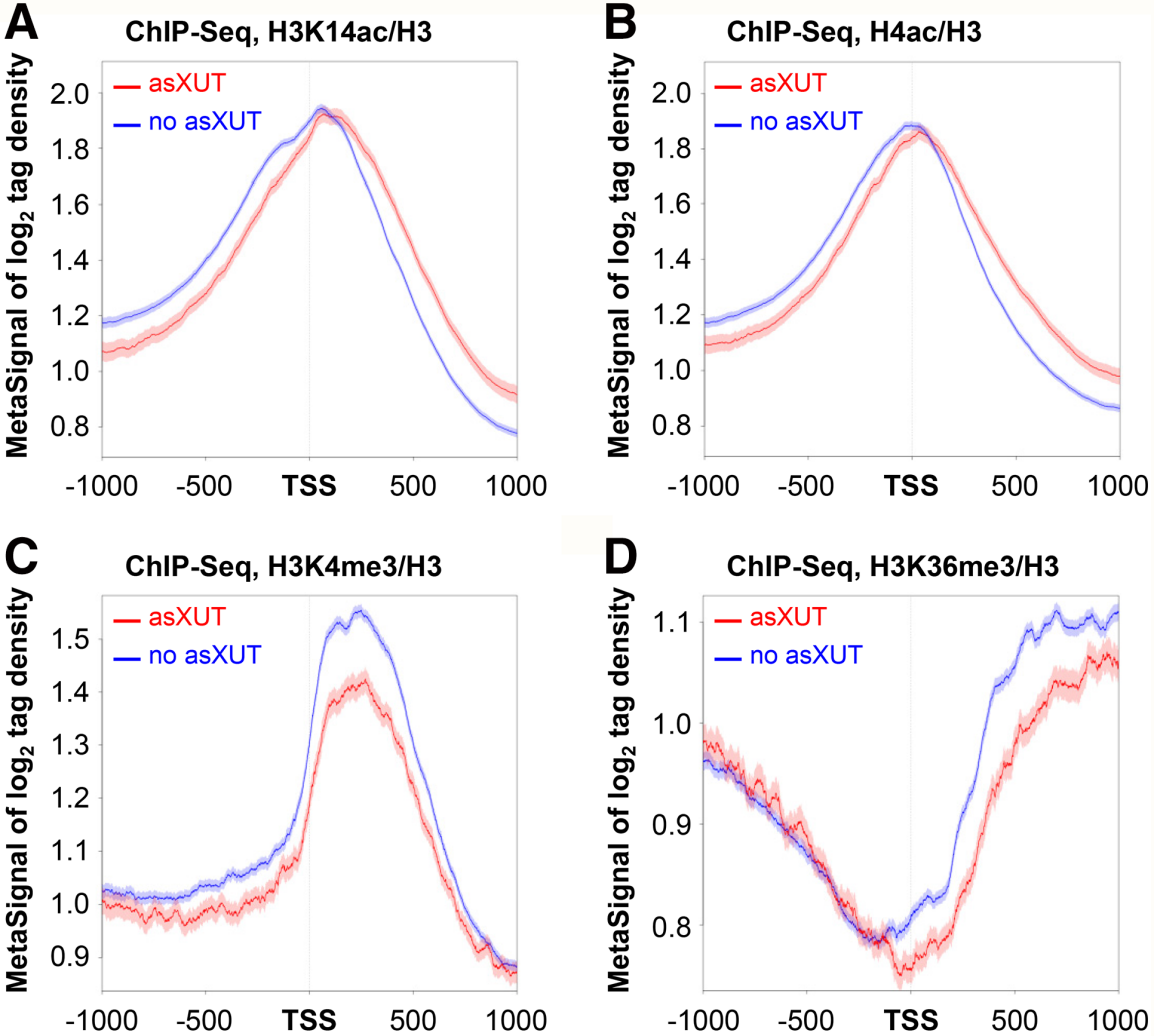
Expression of asXUTs is associated to changes of histone modifications patterns. (*A*) Metagene view of H3K14ac levels in WT cells for genes with (red lines) or without (blue lines) asXUTs. Biological duplicates of YAM2400 (WT) cells were grown to mid-log phase in rich medium. After cross-linking, chromatin extraction and sonication, histone H3 and H3K14ac were immunoprecipitated. Coprecipitated DNA fragments were purified and used to construct ChIP-seq libraries. Metagene representation of signal for each class of genes was performed as above, in a strand-unspecific manner, using ratio of coverage (log_2_) for H3K14ac and H3. The shading surrounding each line denotes the 95% confidence interval. (*B*) Same as above for H4K5/8/12/16 acetylation (H4ac). (*C*) Metagene view of H3K4me3 levels in WT cells for genes with (red lines) or without (blue lines) asXUTs. Analysis was performed as described above, using previously published ChIP-seq data for H3K4me3 and H3 (DeGennaro et al. 2013). Data are presented as above. (*D*) Same as above for H3K36me3, using previously published ChIP-seq data for H3K36me3 and H3 (DeGennaro et al. 2013).

Not surprisingly, according to the negative correlation between sense and antisense transcription described above, asXUT-associated genes in WT cells were significantly less transcribed than those without asXUT (Supplemental Fig. S5A,B). Since active transcription is often correlated with histone acetylation at the promoter of genes (Smolle and Workman 2013), we asked whether promoters of asXUT-associated genes display defects of histone acetylation. To test this, we measured acetylation of histone H3 lysine 14 (H3K14ac) and histone H4 lysines 5/8/12/16 (H4ac) in WT cells using ChIP-seq. We observed reduced H3K14ac and H4ac levels upstream of the promoter of genes with asXUT (Fig. 5A,B), probably reflecting their low transcription. On the other hand, asXUT-associated genes displayed significantly higher levels of H3K14ac and H4ac across the gene body (Fig. 5A,B; see also Supplemental Fig. S5C,D). This is consistent with recent observations showing that H3 acetylation is high along genes with antisense transcription in *S. cerevisiae* (Murray et al. 2015).

Trimethylation of histone H3 lysine 4 (H3K4me3) in the 5′ region of genes and of histone H3 lysine 36 (H3K36me3) across the gene body are two other hallmarks of active transcription (Smolle and Workman 2013; Howe et al. 2017). In budding yeast, genes with antisense transcription display reduced H3K4me3 and H3K36me3 levels (Murray et al. 2015). Using previously published ChIP-seq data (DeGennaro et al. 2013), we observed that genes with asXUTs in fission yeast also display significantly lower levels of H3K4me3 in the 5′ region (Fig. 5C; Supplemental Fig. S5E) and H3K36me3 across the gene body (Fig. 5D; Supplemental Fig. S5F), which might also reflect their low transcription.

Interestingly, we observed similar patterns of histone modifications across the body of the 925 nonconvergent genes for which we detected nascent antisense transcription signal using NET-seq (see Supplemental Fig. 5G–J), reinforcing the idea that antisense transcription is associated to conserved specific histone modifications, and that asXUTs constitute genuine antisense TUs.

Together, these data indicate that asXUTs’ expression in WT cells is associated to several chromatin marks. The observations of similar patterns of histone modifications for genes subject to antisense transcription in budding yeast further suggests a possible conservation across evolution of a signature associated to antisense transcription, at the chromatin level.

### Spt6 represses expression of cryptic antisense XUTs

Spt6 is a conserved histone chaperone that was shown to modulate intragenic and antisense transcription in fission yeast (DeGennaro et al. 2013). In addition, it is required for H3K4me3 and H3K36me3 (DeGennaro et al. 2013). As genes with asXUTs display reduced levels of both histone modifications, we investigated whether asXUTs’ expression is controlled by Spt6.

**FIGURE 6.**
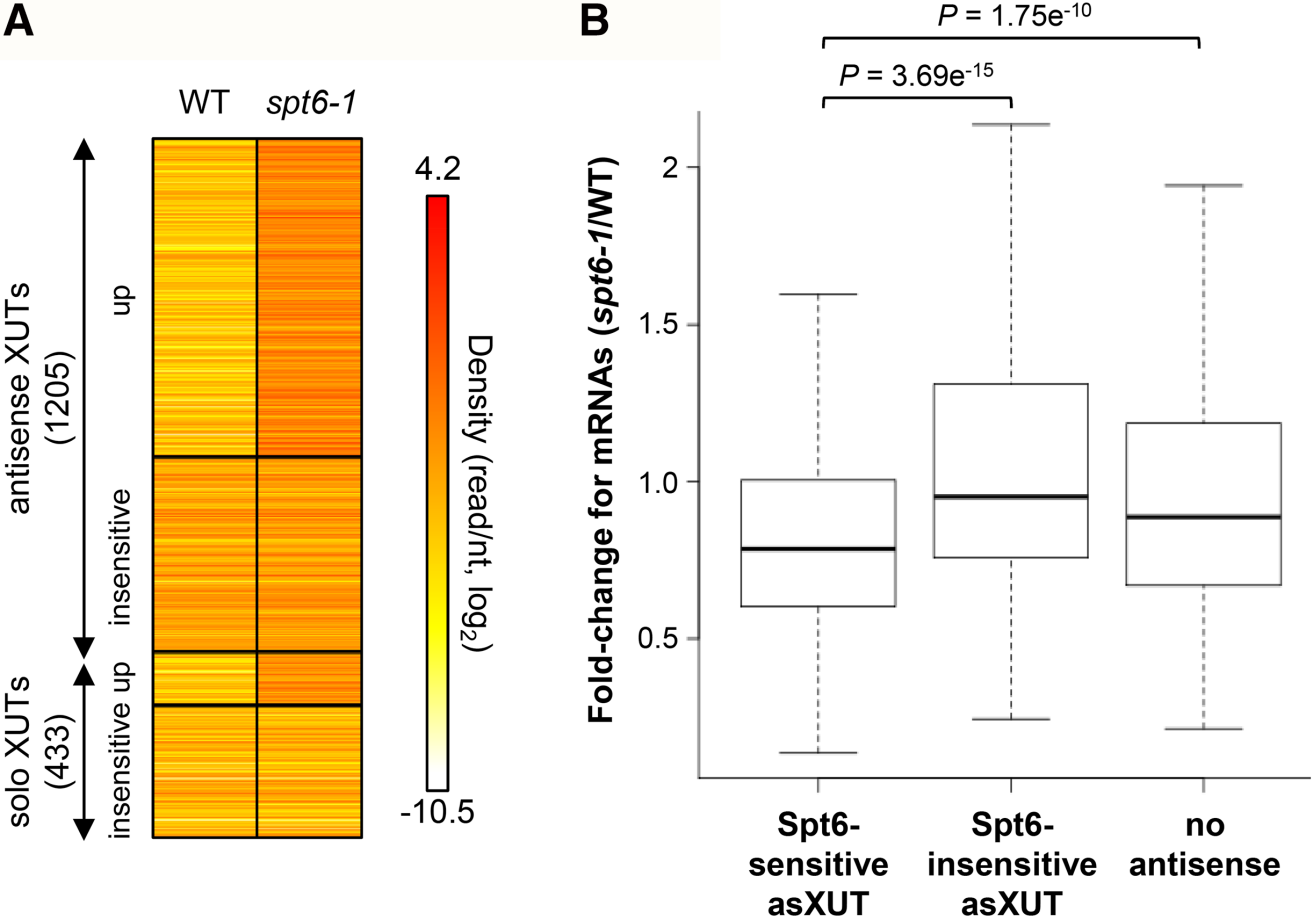
Spt6 represses the expression of a subset of asXUTs. (*A*) XUTs’ accumulation in *spt6-1* cells of *S. pombe*. Densities (tag/nt) for XUTs, computed from previously published poly(A)^+^ RNA-seq data from WT and *spt6-1* cells shifted at 37°C for 2 h (DeGennaro et al. 2013), are shown as a heatmap. Among the 1205 antisense and 433 solo XUTs, 746 and 125 accumulate upon Spt6 inactivation (mutant/WT ratio >2, *P* < 0.05), respectively. (*B*) Effect of Spt6 inactivation on mRNAs. Densities were computed as described above for mRNAs, the asXUT of which accumulated (683) or not (403) in *spt6-1* cells, and for mRNAs without antisense (1221). For each mRNA within each of these categories, the *spt6-1*/WT ratio of density was computed. Data are presented as a box-plot. *P*-values obtained upon Wilcoxon rank-sum test are indicated.

To test this, we analyzed published RNA-seq data from WT and *spt6-1* cells, shifted for 2 h at 37°C (DeGennaro et al. 2013). We observed significant overexpression for 871 XUTs upon heat inactivation of Spt6 (Supplemental Fig. S6A), 746 (86%) of which are antisense (Fig. 6A). This significant enrichment of asXUTs within the set of Spt6-sensitive XUTs (χ^2^test, *P* < 0.00001) is consistent with the role of Spt6 in repressing antisense transcription (DeGennaro et al. 2013). Although it is hazardous to draw any conclusion about transcription from RNA-seq data, we hypothesize that these asXUTs accumulate in *spt6-1* cells due to an increase of their synthesis rather than to a change in their decay, as Exo2 is present and probably fully active in these conditions (otherwise, most XUTs would be expected to accumulate).

Next, we asked whether the overexpression of asXUTs in *spt6-1* cells could be related to any down-regulation on the paired-sense mRNA. Specifically, we observed that mRNAs with a Spt6-sensitive asXUT showed a significantly higher reduction compared to those with a Spt6-insensitive asXUT or without antisense (Fig. 6B; see also Supplemental Fig. S6B). However, for the same reason as mentioned above, it is difficult to determine whether this effect occurs at the transcriptional or post-transcriptional level, and whether it is mediated by a change of asXUT synthesis or by the transcript itself.

In summary, asXUTs’ expression is controlled by Spt6, the loss of which results in the accumulation of a large subset of asXUTs, and this correlates with a down-regulation of the paired-sense mRNA, possibly reflecting a sense/antisense attenuation mechanism.

### A subset of antisense XUTs is up-regulated during meiosis induction

The data presented above showed that XUTs accumulate upon inactivation of Exo2, and also of Spt6 for a subset of them. We asked whether XUTs could also respond to particular environmental conditions.

In budding and fission yeasts, subsets of lncRNAs have been shown to respond to sexual differentiation (Bitton et al. 2011; Lardenois et al. 2011). To test whether this is also the case for XUTs in *S. pombe*, we analyzed published RNA-seq data from *pat1-114* diploid cells (mutated for the meiotic inhibitor Pat1), collected before and after 2, 4, 6, and 8 h of meiosis induction (Bitton et al. 2015). We observed significant accumulation (greater than twofold enrichment and *P* < 0.05) for 724 XUTs, in at least one time point (Supplemental Fig. S7A,B). Among these XUTs, 561 are antisense and 441 correspond to novel transcripts, not annotated before this work.

**FIGURE 7.**
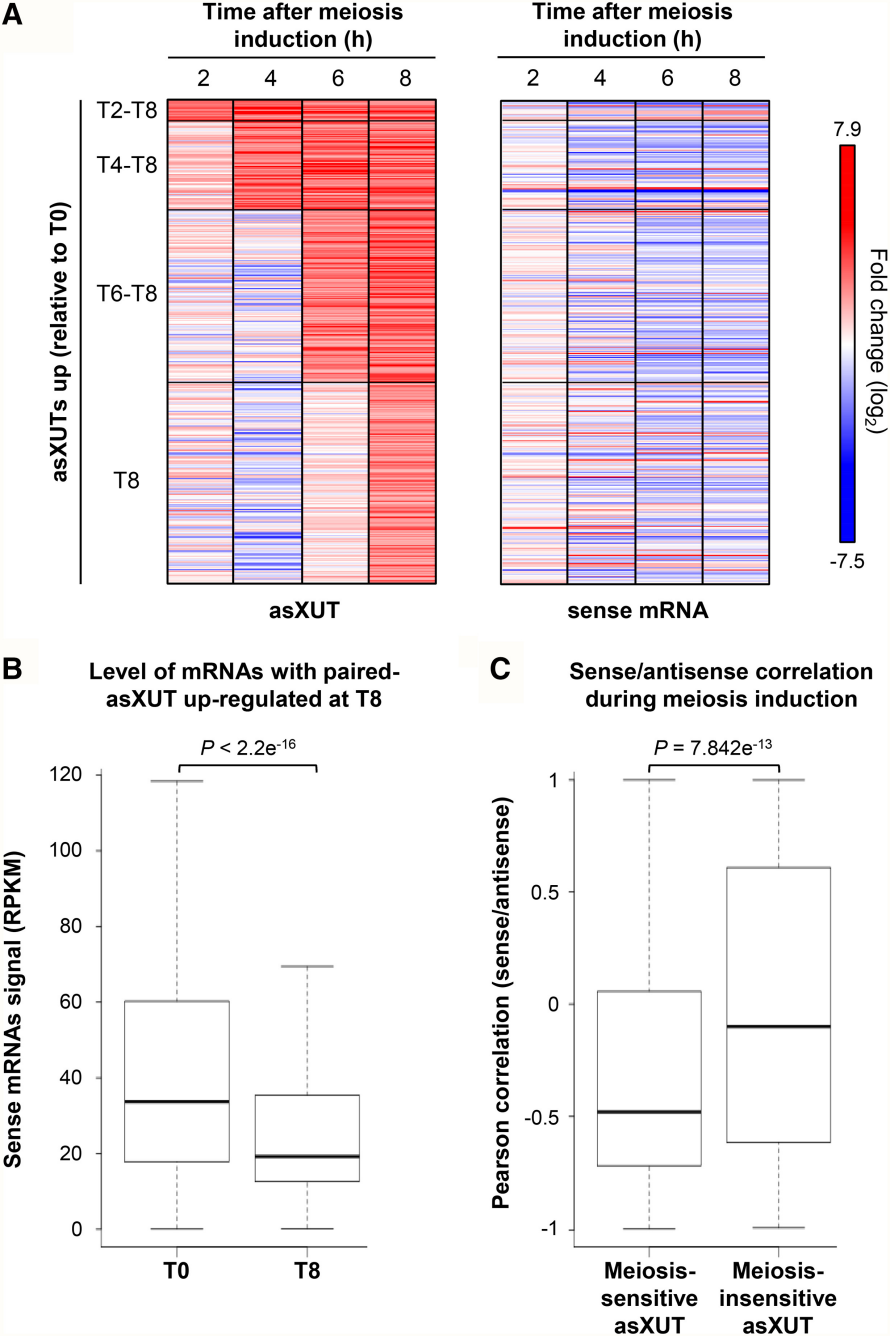
Overexpression of meiosis-responsive asXUTs correlates with paired-sense mRNA down-regulation during meiosis induction. (*A*) Signal (RPKM) for asXUTs and sense mRNAs in *pat1-114* diploid cells collected before (T0) or after 2, 4, 6, or 8 h of meiosis induction (T2-8) were computed from previously published RNA-seq data (Bitton et al. 2015). From these data, we selected 453 asXUTs showing significant accumulation at least at T8 (T8/T0 fold-change >2, *P* < 0.05). For each of these asXUTs and their paired-sense mRNAs, the fold-change relative to T0 was computed for each time point. Data are presented as heatmaps. (*B*) Box-plot of signal (RPKM) at T0 and T8 for mRNAs with significantly up-regulated paired-asXUT at T8. The *P*-value obtained upon Wilcoxon rank-sum test is indicated. (*C*) Box-plot of Pearson's correlation coefficients for mRNA/asXUT pairs during meiosis induction. For each pair of mRNA/asXUT, a Pearson's correlation coefficient was computed from the signal (RPKM) for the mRNA and its paired-asXUT, at each time point. The distribution of the correlation coefficient was then analyzed as a box-plot for mRNAs with a meiosis-responsive asXUT (525) or with a meiosis-insensitive asXUT (561). The *P*-value obtained upon Wilcoxon rank-sum test is indicated.

Importantly, overexpression of meiosis-sensitive asXUTs correlated with paired-sense mRNAs’ down-regulation. First, we observed a global down-regulation of mRNAs for which the paired-asXUTs accumulated at least after 8 h of meiosis induction (median T8/T0 fold-change = 0.61; Fig. 7A,B). Second, using the signal (RPKM) for each asXUT and its paired-sense mRNA at each time point during meiosis induction, we determined the Pearson's correlation coefficient for each pair of asXUT/mRNA (for an illustrative example, see Supplemental Fig. S7C,D). We observed a significant difference in asXUT/mRNA correlations depending on whether the asXUT responded to meiosis induction or not (Fig. 7C, *P* = 7.842 × 10^−13^, Wilcoxon rank-sum test), with asXUTs globally anti-correlated to sense mRNA levels when the asXUT was meiosis-responsive (median = −0.48), but not when it was unaffected (median = −0.10).

Thus, XUTs respond to meiosis induction, and overexpression of meiosis-responsive asXUTs correlates with global paired-sense down-regulation, providing supportive evidence that sense/antisense modulation is dynamic and is a general property of the majority of meiotic genes.

## DISCUSSION

In this work, we combined nascent transcription measurement with cryptic aslncRNAs profiling. We revealed that antisense transcription in fission yeast concerns the majority of protein-coding genes and correlates with low sense expression. We propose that antisense transcription affects sense transcription directly in *cis*, and also in *trans* through regulatory aslncRNAs.

Identification of aslncRNAs using RNA-seq or microarray has provided evidence for antisense transcription in many organisms, including fission yeast. However, these approaches based on mature RNA detection underestimate the accurate extent of antisense transcription. Here, using NET-seq, which currently constitutes the state of the art technique to measure nascent transcription per se, independently of post-transcriptional RNA degradation, in a strand-specific manner (Churchman and Weissman 2011), we detected an antisense transcription signal for 68% of protein-coding genes in fission yeast. Cumulatively, combining the evidence at the transcription (NET-seq) and/or at the RNA level (annotated aslncRNA/asXUT), antisense transcription could concern up to 3905 genes (76.9%) in fission yeast (see Supplemental Fig. S3B), considerably extending the current antisense catalog.

What is the effect of antisense transcription? We observed a global anti-correlation relationship with sense transcription, genes with antisense being significantly less transcribed than genes without antisense. Strikingly, levels of sense transcription decrease as the overlap by the nascent antisense transcription signal increases. This observation is consistent with previous measurements of sense mRNA synthesis using RNA labeling (Eser et al. 2016). In addition, among all the genes displaying antisense transcription, we noted that those for which the antisense signal overlaps the TSS are significantly less transcribed. Interestingly, previous microarray analyses in budding yeast have shown that genes with TSS overlapped by antisense transcripts display more variable expression (Xu et al. 2011). However, this conclusion based on steady-state RNA measurement would be impacted by additional parameters such as RNA (de)stabilization or trafficking, possibly linked to dsRNA formation. Here, our data not only reinforce this idea that the overlap of the sense TSS is a key determinant for the antisense-associated regulation of sense gene expression but provide evidence that the effect occurs at the level of nascent transcription. Similar observations have been recently made in mammalian cells (Mayer et al. 2015; Chen et al. 2016), suggesting that the underlying mechanism could be conserved across evolution.

At the RNA level, the produced aslncRNAs appeared to be cryptic transcripts, mainly degraded by the Xrn1/Exo2-dependent 5′–3′ cytoplasmic RNA decay pathway. Inactivation of Exo2 resulted in the stabilization of 1638 XUTs, including 815 novel and 1205 antisense transcripts. Combined to the current annotation in *S. pombe*, this would give at least 2824 annotated lncRNAs, with 1895 antisense, among which 1205 are XUTs. However, it is possible that the number of aslncRNAs is still underestimated since some of them might be targeted by RNA decay machineries other than Exo2, such as the nuclear exosome. Consistent with this idea, 309 of the 461 genes for which the antisense transcription signal could not be attributed to a convergent TU or an annotated aslncRNA/asXUT displayed an antisense RNA signal sensitive to Rrp6, which might correspond to CUTs.

XUTs are cryptic transcripts targeted by Xrn1/Exo2. One key point is to determine whether they can accumulate in biological conditions other than mutants defective for Xrn1/Exo2. If so, of particular importance would be to show any paired-sense gene regulation associated to this accumulation. In this regard, we defined a set of Spt6-sensitive asXUTs that accumulated upon inactivation of the conserved histone chaperone Spt6. More importantly, we identified a subset of asXUTs accumulated during meiosis induction. In both cases, asXUT accumulation correlated with paired-sense gene down-regulation. However, in the absence of NET-seq data that could allow precisely addressing the levels of sense and antisense transcription in these conditions, we cannot exclude that the observed down-regulation of sense mRNA levels results, at least in part, from a *cis* effect (i.e., increase of asXUT transcription) rather than from an asXUT-mediated effect in *trans*.

A key question for aslncRNAs is to determine their relationship with RNAi. Several examples of aslncRNAs have been described to control the paired-sense gene transcription independently of siRNA in plants (Swiezewski et al. 2009) and mammals (Yu et al. 2008), indicating that regulatory aslncRNAs can escape the nuclear and cytoplasmic RNAi pathways. Fission yeast is an excellent model for RNAi-mediated transcriptional gene silencing, and extensive studies have described the mechanism of action and the interplay with chromatin marks. Here we show that asXUTs are not targeted by RNAi in *S. pombe*. One possibility is that asXUTs and paired-sense mRNAs do not coexist within the same cells, or do not form dsRNA. In *S. cerevisiae*, sense/antisense pairing has been debated, as it was first reported that sense and antisense transcripts never coexist in the same cell (Castelnuovo et al. 2013). However, recent studies confirmed that although transcribed in a bimodal fashion, sense and antisense RNAs can coexist within the same cell (Nguyen et al. 2014; Lenstra et al. 2015) and form dsRNA structures in vivo (Drinnenberg et al. 2011; Sinturel et al. 2015; Wery et al. 2016). In *S. pombe*, the most likely explanation is that Dicer and the asXUT/mRNA duplexes are prevented for interaction due to different subcellular localization. This would fit with the idea that Exo2 acts in the cytoplasm while Dicer is restricted to the nucleus and localizes to specific chromosomal regions, mainly the centromeric repeats (Woolcock and Bühler 2013). Further work will be required to determine whether asXUTs can also form dsRNA in vivo in fission yeast, and whether this is important for the regulation of paired-sense genes.

In conclusion, our work revealed that antisense transcription in fission yeast is much more extended than previously expected, and contributes to regulate gene expression, in *cis* but also probably in *trans*, through the production of regulatory aslncRNAs. We propose that antisense transcription constitutes a widespread nonpromoter determinant for fine-tuning of gene expression. The cytoplasmic 5′-end RNA decay plays a key role in controlling aslncRNAs, from budding to fission yeasts. The high conservation of Xrn1 in the eukaryotic tree opens the fundamental questions of the existence of asXUT and of their regulatory activity in higher eukaryotes, adding an additional layer to gene regulation complexity.

## MATERIALS AND METHODS

### Yeast strains and media

Strains YAM2394 (*h^+^ ade6-M210 ura4-D18 leu1-32*) and YAM2397 (*h^+^ ade6-M210 ura4-D18 leu1-32 exo2Δ::kanMX4*) were purchased from Bioneer and verified by PCR on genomic DNA and northern blot. Strain YAM2402 (*h^−^ exo2Δ::kanMX4*) was constructed by deleting *exo2* in the YAM2400 (*h*^−^), then verified by PCR on genomic DNA. Strain YAM2492 (*h^−^ rpb3-flag::natMX6*) was constructed by transformation, then verified by PCR on genomic DNA and western blot. All strains were grown at 32°C to mid-log phase in YES medium (MP Biomedicals).

### NET-seq

NET-seq analysis was performed from two biological replicates of strain YAM2492 (*rpb3-flag*). Libraries were constructed as previously described (Churchman and Weissman 2011, 2012; Marquardt et al. 2014), starting from 1 L of exponentially growing (OD_595_ 0.7–0.8) cells. RNAPII was immunoprecipitated using anti-FLAG M2 affinity gel (Sigma-Aldrich), then recovered upon two successive elution steps using FLAG peptide (Sigma). Ligation of DNA 3′-linker was performed from 2–3 µg of purified nascent RNA (i.e., coimmunoprecipitated with RNAPII; IP samples) and total RNA (input samples). Ligated RNAs were then submitted to alkaline fragmentation for 20 min at 95°C. Single-end sequencing (50 nt) of the libraries was performed on a HiSeq 2500 sequencer, giving between 20 and 50 million reads. After trimming of the 5′-adapter, reads were uniquely mapped to the reference genome (ASM294v2.30) using version 0.12.8 of Bowtie (Langmead et al. 2009), with a tolerance of two mismatches, giving 7.1–9.8 million uniquely mapped reads.

Data were normalized on the total number of uniquely mapped reads, for each sample. As a reference for absence of transcription, we used a set of 50 protein-coding genes, showing the lowest nascent transcription signals (IP samples) and low total RNA levels (input samples). These genes were selected after filtering out the genes for which the signal was equal or close to zero as no (or very few) reads could be uniquely mapped, due to the high content of repetitive sequences. This was manually checked using the NET-seq signals (IP and input) and also the ChIP-seq signals (IP) for histone H3.

Snapshots were produced using the VING software (Descrimes et al. 2015).

### Analysis of convergent TU

A gene was considered as convergent to another TU [protein-coding gene, sn(o)RNA, tRNA, lncRNA] when (i) the distance between its annotated 3′ coordinate and the convergent TU one was <500 bp, and (ii) the nascent transcription signals overlap on ≥50 nt with a coverage ≥1 read/nt per strand, in each biological replicate.

### Total RNA extraction

Total RNA was extracted from exponentially growing cells using standard hot phenol procedure, resuspended in nuclease-free H_2_O (Ambion) and quantified using a NanoDrop 2000c spectrophotometer. Quality and integrity of extracted RNA was checked by analysis in an RNA 6000 Pico chip in a 2100 bioanalyzer (Agilent).

### Northern blot

Ten micrograms of total RNA were loaded on denaturing 1.2% agarose gel and transferred to Hybond-XL nylon membrane (GE Healthcare). ^32^P-labeled oligonucleotide probes (listed in Supplemental Table S1) were hybridized overnight at 42°C in ULTRAhyb-Oligo hybridization buffer (Ambion).

### RT-qPCR

Strand-specific reverse transcription (RT) reactions were performed from at least three biological replicates, using 1 µg of total RNA and the SuperScriptII Reverse Transcriptase Kit (Invitrogen), in the presence of 6.25 µg/mL actinomycin D. For each sample, a control without RT was included. Subsequent quantitative real-time PCR was performed on technical duplicates, using a LightCycler 480 instrument (Roche). Oligonucleotides used are listed in Supplemental Table S1.

### Total RNA-seq

Total RNA-seq analysis was performed from two biological replicates of the YAM2394 (WT) and YAM2397 (*exo2*Δ) strains. Ribosomal RNAs were depleted from total RNA using the RiboMinus Eukaryote v2 Kit (Life Technologies). Depletion efficiency and quality of rRNA-depleted RNA was assessed by analysis in RNA Pico 6000 chip for 2100 Bioanalyzer (Agilent).

Total RNA-seq libraries were prepared from 125 ng of rRNA-depleted RNA using the TruSeq Stranded Total RNA Sample Preparation Kit (Illumina). Paired-end sequencing (2 × 50 nt) was performed on a HiSeq 2500 sequencer. Reads were mapped using version 2.0.6 of TopHat, with a tolerance of three mismatches and a maximum size for introns of 2 kb. Tag densities were normalized on sn(o)RNA levels for all subsequent analyses.

### Annotation of XUTs

Segmentation was performed using the ZINAR algorithm (Wery et al. 2016). Briefly, the uniquely mapped reads from the *exo2*Δ biological duplicates were pooled. A strand-specific signal was then computed for each nucleotide as the number of times it is covered by a read or the insert between two paired reads. After log_2_ transformation, the signal was smoothed using a 10–200-nt sliding window (with 10-nt increments). All genomic regions showing a smoothed log_2_ signal value above a threshold (ranging from 1.44 to 216, with 1.44 increments) were reported as segments. XUTs were defined as ≥200 nt segments that do not overlap ORF and that show a greater than twofold enrichment in *exo2Δ* vs WT, with a *P*-value (adjusted for multiple testing with the Benjamini–Hochberg procedure) <0.05 upon differential expression analysis using DESeq (Anders and Huber 2010). In total, 3000 segmentations with different sliding window size and threshold parameters were tested, among which we selected the one showing the best compromise between XUT and mRNA detection. A XUT was reported as antisense when the overlap with the sense mRNA was ≥50 nt.

### Identification of Spt6-sensitive and meiosis-responsive XUTs

Previously published poly(A)^+^ RNA-seq data from WT and *spt6-1* cells shifted at 37°C for 2 h (DeGennaro et al. 2013), and from *pat1-114* diploid cells collected before or after 2, 4, 6, or 8 h of meiosis induction (Bitton et al. 2015) were retrieved from the NCBI Gene Expression Omnibus (accession number: GSE49573) and from the NCBI BioProject (accession number: PRJEB7403, samples ERS555597-606), respectively. Differential expression analyses were performed using DESeq (Anders and Huber 2010) upon normalization on the total number of uniquely mapped reads. Up-regulated XUTs were defined on the basis of a minimum twofold enrichment and a *P*-value <0.05 (adjusted for multiple testing with the Benjamini–Hochberg method).

### Small RNA-seq

Small RNA-seq libraries from YAM2394 (WT) and YAM2397 (*exo2*Δ) strains were constructed according to the Small RNA Sample Preparation Guide (Illumina) using 10- to 40-nt small RNAs purified from total RNA on 15% TBE–urea PAGE. Single-end sequencing (40 nt) of libraries was performed on a Genome Analyzer IIx (Illumina). After adapter sequences trimming, reads were uniquely mapped using the version 0.12.8 of Bowtie (Langmead et al. 2009), with a tolerance of three mismatches. Mapping on centromeric repeats was performed using the version 0.12.9 of Bowtie, with a three mismatches tolerance, reporting all alignments per read.

### ChIP-seq

ChIP-seq analysis was performed from two biological replicates of YAM2394 (WT) cells. Briefly, exponentially growing (OD_595_ 0.5) cells were fixed for 10 min at room temperature using formaldehyde (1% final concentration), then glycine was added (0.4 M final concentration). Histone H3, H3K14ac and H4K5/8/12/16ac were immunoprecipitated using ab1791 (Abcam), 07-353 (Millipore), and 05-1355 (Millipore) antibodies, respectively.

Libraries were prepared from 5 ng of “input” or “IP” DNA using the TruSeq ChIP Sample Preparation Kit (Illumina). Single-end sequencing (50 nt) was performed on a HiSeq 2500 sequencer. Reads were uniquely mapped using version 2.1.0 of Bowtie2 (Langmead and Salzberg 2012), with a tolerance of one mismatch in seed alignment.

### Statistical tests

The Wilcoxon rank-sum test was used for the analysis of data sets showing non-normal distribution (normality was assessed using the Shapiro–Wilk test). The *t*-test was used for analysis of RT-qPCR data.

## DATA DEPOSITION

Raw sequences have been deposited to the NCBI Gene Expression Omnibus (accession number GEO: GSE72382). Accession numbers for previously published RNA-seq data are GSE64992 (*rrp6*), GSE49573 (*spt6*), and PRJEB7403 (meiosis; sample accession ERS555597-606). Genome browsers for visualization of processed data are publicly accessible at http://vm-gb.curie.fr/mw1.

## SUPPLEMENTAL MATERIAL

Supplemental material is available for this article.

## Supplementary Material

Supplemental Material
