# Supplementary material for: Native elongating transcript sequencing reveals global anti-correlation between sense and antisense nascent transcription in fission yeast
Source: RNA. 2018 Feb;24(2):196–208. doi: 10.1261/rna.063446.117 (PMC5769747; doi:10.1261/rna.063446.117)
Supplement: Supplemental Material [file supp_24_2_196__index.html]

Native elongating transcript sequencing reveals global anti-correlation between sense and antisense nascent transcription in fission yeast — Supplemental Material 

# Native elongating transcript sequencing reveals global anti-correlation between sense and antisense nascent transcription in fission yeast

## Supplemental Material

- Supplemental\_Figure\_Legends.docx
- Supplemental\_Table\_S1.pdf
- Supplemental\_Figures.pdf
