# Supplementary material for: Native elongating transcript sequencing reveals global anti-correlation between sense and antisense nascent transcription in fission yeast
Source: RNA. 2018 Feb;24(2):196–208. doi: 10.1261/rna.063446.117 (PMC5769747; doi:10.1261/rna.063446.117)
Supplement: Supplemental Material [file supp_063446.117_Supplemental_Figure_Legends.docx]

**SUPPLEMENTAL FIGURE LEGENDS**

**Supplemental Figure S1. Extensive antisense transcription in fission yeast.**

(***A***) RNAPII purification for construction of NET-Seq libraries. Total extracts (T) were prepared from two biological replicates of WT (YAM2492) cells, digested with DNase I then mixed with anti-FLAG affinity gel. After incubation at 4°C, supernatant (S) containing unbound material was discarded. After washes, the immunoprecipitated (IP) material was eluted using FLAG peptide. Aliquots of T, IP, S and G (post-elution gel containing not eluted material) fractions were analyzed by Western-blot using mouse anti-FLAG M2 antibody. (***B***) Snapshot of total RNA (input) and nascent transcript (IP) signals along the *act1* gene in WT cells. In each panel, the signal corresponding to the + and – strand is shown in blue and pink, respectively. Blue arrow and box represent the mRNA and coding sequences, respectively. Grey arrows represent two novel ncTU (Eser et al. 2016). The region beyond the polyadenylation site of *act1* is highlighted using a green box. The snapshot was produced using VING (Descrimes et al. 2015). (***C***) Box-plot of nascent transcription (NET-Seq, IP samples) signals (tag/nt, log_2_) for exon and intron regions of intron-containing genes. (***D***) Same as above for total RNA (NET-Seq, input samples) signals (tag/nt, log_2_). (***E***) Scatter-plot showing NET-Seq signals (tag/nt, log_2_ scale) for mRNAs in the two biological replicates of WT (YAM2492) cells. The Pearson’s correlation coefficient (ρ) is indicated. (***F***) Same as above for annotated ncRNAs, corresponding to the 1522 ncRNAs from Pombase plus 487 novel ncTUs (Eser et al. 2016). (***G***) Scatter-plot showing NET-Seq nascent transcription (IP) and total RNA (input) signals (RPKM) for the set of 50 ‘untranscribed’ protein-coding genes (black), the other protein-coding genes (blue) and ncRNAs (green). (***H***) Box-plot of nascent transcript signal (RPKM, log_2_) in WT cells for the antisense of the 3455 protein-coding genes with antisense (magenta), for the 1522 ncRNAs from Pombase (light green) and for the 487 novel ncTUs (dark green) (Eser et al. 2016). (***I***) Same as above for coverage (percentage of nt covered by ≥1 uniquely mapped read, in each of the two biological replicates).

**Supplemental Figure S2. Sense transcription decreases with the overlap by antisense transcription.**

(***A***) Metagene view of total RNA signal (NET-Seq, input) for genes without antisense transcription (1624; black), genes with overlapping convergent TU (2530; blue) and genes with antisense but not convergent transcription (925; red). Sense and antisense meta-signals were computed and plotted as described in Fig. 1F. The shading surrounding each line denotes the 95% confidence interval. (***B***) Box-plot of NET-Seq signal (tag/nt, log_2_ scale) in WT cells for genes with increasing coverage by antisense transcription. The 3455 genes with significant antisense transcription were divided into five classes according to the coverage by antisense signal. *P*-values obtained upon Wilcoxon rank-sum test are indicated. (***C***) Same as above for sense/antisense NET-Seq signal (tag/nt) ratio (log_2_ scale). (***D***) Metagene view of sense nascent transcription signal for genes for which antisense nascent transcription overlaps the TSS (279; dark grey) or not (3176; light grey). The shading surrounding each line denotes the 95% confidence interval. (***E***) Venn diagram showing the genes with antisense transcription (3455), with a convergent overlapping TU (2530), and with a previously annotated aslncRNA (1286).

**Supplemental Figure S3. XUT lncRNAs landscape in fission yeast.**

(***A***) Scatter plot of tag density for mRNAs (grey), sn(o)RNAs (black) and XUTs (red) in WT and *exo2Δ,* computed using uniquely mapped reads. Results are presented as log_2_ of density, expressed in tag/nt. The black dashed line indicates no change (*exo2Δ*/WT ratio = 1). (***B***) Venn diagram showing the genes with antisense transcription (3455), with a convergent overlapping TU (2530), with a previously annotated aslncRNA (1286), and with asXUT (1086). (***C***) RT-qPCR analysis of *XUT1322* and *puf5* mRNA levels in WT and *exo2Δ *cells. Strains YAM2400 (WT) and YAM2402 (*exo2Δ*) were grown in rich YES medium to mid-log-phase. Transcripts levels were determined by strand-specific RT-qPCR from total RNA and normalized on U3B snoRNA level. Data are presented as mean +/- standard deviation (SD), calculated from four biological replicates. ^***^ *p* < 0.001; ^**^ *p* < 0.01 upon t-test. (***D***) Same as above for *XUT0194* and *cdt1* mRNA. (***E***) Same as above for *XUT0647* and *rev3* mRNA. (***F***) Same as above for *XUT0433* and *SPBC8E4.05c* mRNA. (***G***) Snapshot of total RNA-Seq signal in WT and *exo2Δ* cells along the *XUT1322*/*puf5* pair*.* Signals for the + and - strands are visualized as heatmaps in the upper and lower panels, respectively, using the VING software (Descrimes et al. 2015) The stars indicate the position of the oligonucleotides used for the strand-specific RT and then for the qPCR. (***H***) Same as above for the *XUT0194*/*cdt1* pair*.* (***I***) Same as above for the *XUT0647*/*rev3* pair.(***J***) Same as above for the *XUT0433*/*SPBC8E4.058c* pair*.*

**Supplemental Figure S4. Antisense XUTs are insulated from RNAi in fission yeast.**

(***A***) Box-plot of densities (tag/nt, linear scale) of 18-30 nt small RNAs mapped on the ‘overlap’ region or the ‘solo’ region of mRNAs with asXUTs, in WT (dark grey) and *exo2Δ* (light grey). (***B***) Size distribution of small RNAs mapped on the ‘overlap’ region of mRNAs with asXUTs, in WT and *exo2Δ*. (***C***) Size distribution of small RNAs mapped on the ‘solo’ region of mRNAs with asXUTs, in WT and *exo2Δ*. (***D***) Size distribution of small RNAs mapped to the centromeric repeats in WT and *exo2Δ*. (***E***) Composite view of 20-24 nt small RNAs mapped along chromosome III in WT and *exo2Δ* . Signal for the + and - strand is shown in blue and pink, respectively. Position of the centromere is indicated by the light grey sphere.

**Supplemental Figure S5. Expression of asXUTs is associated to changes of histone modifications patterns.**

(***A***) Metagene view of NET-Seq signals along genes with (red lines) or without (blue lines) asXUT, in WT cells. For each class of genes, normalized coverage (tag/nt, log_2_) along mRNA transcription start site (TSS) +/- 1000 nt (+ strand) and the antisense strand (- strand) were piled up, in a strand-specific manner, and average signals for the + and - strands were plotted. The shading surrounding each line denotes the 95% confidence interval. (***B***) Box-plot of nascent transcription (NET-Seq) signal for genes with (red) or without (blue) asXUT in WT cells. The *P*-value obtained upon Wilcoxon rank-sum test is indicated. (***C***) Box-plot of H3K14ac levels along the body (TSS to TTS) of genes with (red) or without (blue) asXUT, in WT cells. Preparation of ChIP-Seq libraries, analysis and normalization (on H3 levels) of the data are described in Fig. 5A. The *P*-value obtained upon Wilcoxon rank-sum test is indicated. (***D***) Same as above for H4ac levels. (***E***) Box-plot of H3K4me3 levels along the TSS to +500 region of genes with (red) or without (blue) asXUT, in WT cells. Previously published ChIP-Seq data (DeGennaro et al. 2013) were analyzed and normalization as in Fig. 5C. The *P*-value obtained upon Wilcoxon rank-sum test is indicated. (***F***) Box-plot of H3K36me3 levels along the body (TSS to TTS) of genes with (red) or without (blue) asXUT, in WT cells. Previously published ChIP-Seq data (DeGennaro et al. 2013) were analyzed and normalization as in Fig. 5D. The *P*-value obtained upon Wilcoxon rank-sum test is indicated. (***G-J***) Same as (***C-F***), respectively, for the 1624 genes without nascent antisense transcription (black) and the 925 genes with ‘other’ (non convergent) antisense (red), defined in Fig. 2A.

**Supplemental Figure S6. Spt6 inactivation results into the accumulation of a subset of asXUT.**

(***A***) Scatter plot of tag density (log_2_ scale) for mRNAs (grey) and XUTs (red) in WT and *spt6-1* cells shifted for 2h at 37°C*.* Densities were computed from previously published RNA-Seq data (DeGennaro et al. 2013). The black dashed line indicates a 2-fold increase. (***B***) Box-plot of densities (tag/nt, log_2_) for mRNAs in WT (black) and *spt6-1* (red) cells shifted for 2h at 37°C*.* Three subgroups of mRNAs are analyzed: with Spt6-sensitive asXUT (n=683), with Spt6-insensitive asXUT (n=403), without antisense (n=1221).

**Supplemental Figure S7. Accumulation of XUTs upon meiosis induction.**

(***A***) Heatmap visualization of XUTs stabilization upon meiosis induction in WT cells. Signal (RPKM) for XUTs in cells collected before (T0) or after 2, 4, 6 or 8 hours of meiosis induction (T2-8) were computed from previously published RNA-Seq data (Bitton et al. 2015). XUTs are divided into 8 subgroups: XUTs that accumulate at T2-8 (a; n=22), at T4-8 (b; n=105), at T6-8 (c; n=201), at T2 only (e; n=18), at T4 only (f; n=66), at T6 only (g; n=21) or at T8 only (h; n=234). Subgroup d (n=57) corresponds to XUTs that accumulate in ≥2 other time points. (***B***) Venn diagram showing the XUTs accumulated (T_i_/T0 ratio >2, *P*<0.05) after 2h (64), 4h (240), 6h (370) or 8h (595) of meiosis induction. (***C***) Levels (RPKM) for the *slu7* mRNA (blue) and the paired antisense *XUT1122* (red), before or after 2, 4, 6 or 8 hours of meiosis induction. (***D***) Anti-correlation between *slu7* mRNA and *XUT1122* levels during meiosis induction. Levels (RPKM) of each transcript after 2, 4, 6 or 8 hours of meiosis induction were plotted. A tendency curve and the Pearson’s correlation coefficient (ρ) were computed.
