# Supplementary material for: Native elongating transcript sequencing reveals global anti-correlation between sense and antisense nascent transcription in fission yeast
Source: RNA. 2018 Feb;24(2):196–208. doi: 10.1261/rna.063446.117 (PMC5769747; doi:10.1261/rna.063446.117)
Supplement: Supplemental Material [file supp_063446.117_Supplemental_Table_S1.pdf]

Supplemental Table S1. **Oligonucleotides.**

ID	Sequence 5'-3'	Target	Use
AMO1978	CTAAACATCAAAGCTAACGCACCCTC	*exo2*	Northern blot
AMO1986	AAACAGCCGCATTGGGATGGAAGCG	*XUT0444/gal1*	RT (*XUT0444*) & Northern-blot
AMO1990	TACACATGCACGGTCCTTTG	*XUT0444/gal1*	RT (*gal1*) & qPCR
AMO1991	GAGCTGGCTAAGGAATTAGGG	*XUT0444/gal1*	qPCR
AMO2080	CTTTGAGGATGTGCTGATCG	U3B	qPCR
AMO2081	CATGACACGACCAAAAGGAA	U3B	Northern-blot, RT & qPCR
AMO3210	CGAAAGAAGCCGTTTCAGAG	*XUT0647/rev3*	RT (*XUT0647*) & qPCR
AMO3211	TTTAGTGGGATCTGGGGTCA	*XUT0647/rev3*	RT (*rev3*) & qPCR
AMO3214	ACCAAACCAACCTTTGTGCT	*XUT0194/cdt1*	RT (*XUT0194*) & qPCR
AMO3215	CAACACGCTTTCCTTGATGA	*XUT0194/cdt1*	RT (*cdt1*) & qPCR
AMO3219	CAAGATGTGACGGACAGTGC	*XUT0433/SPBC8E4.05c*	RT (*XUT0433*) & qPCR
AMO3220	ATTGGAACCGCCTGTTGTAG	*XUT0433/SPBC8E4.05c*	RT (*SPBC8E4.05c*) & qPCR
AMO2323	AGGAAACGCTTCTTTCAGCA	*XUT1322/puf5*	RT (*XUT1322*) & qPCR
AMO2324	TGTGGGAAGACGAAATAGCA	*XUT1322/puf5*	RT (*puf5*) & qPCR
