# Supplementary figures and images for: Native elongating transcript sequencing reveals global anti-correlation between sense and antisense nascent transcription in fission yeast
Source: RNA. 2018 Feb;24(2):196–208. doi: 10.1261/rna.063446.117 (PMC5769747; doi:10.1261/rna.063446.117)

Supplemental Fig. S1

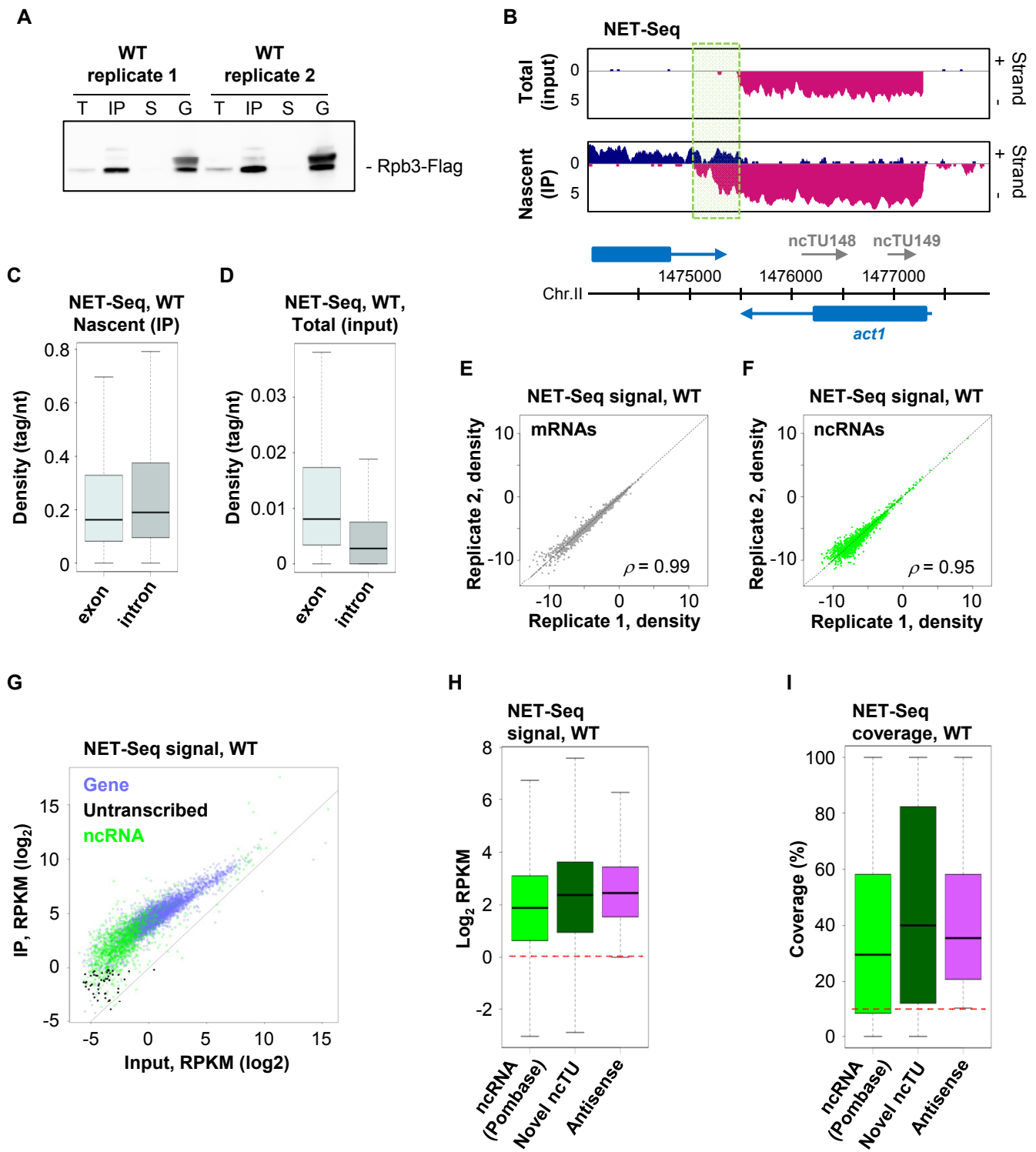

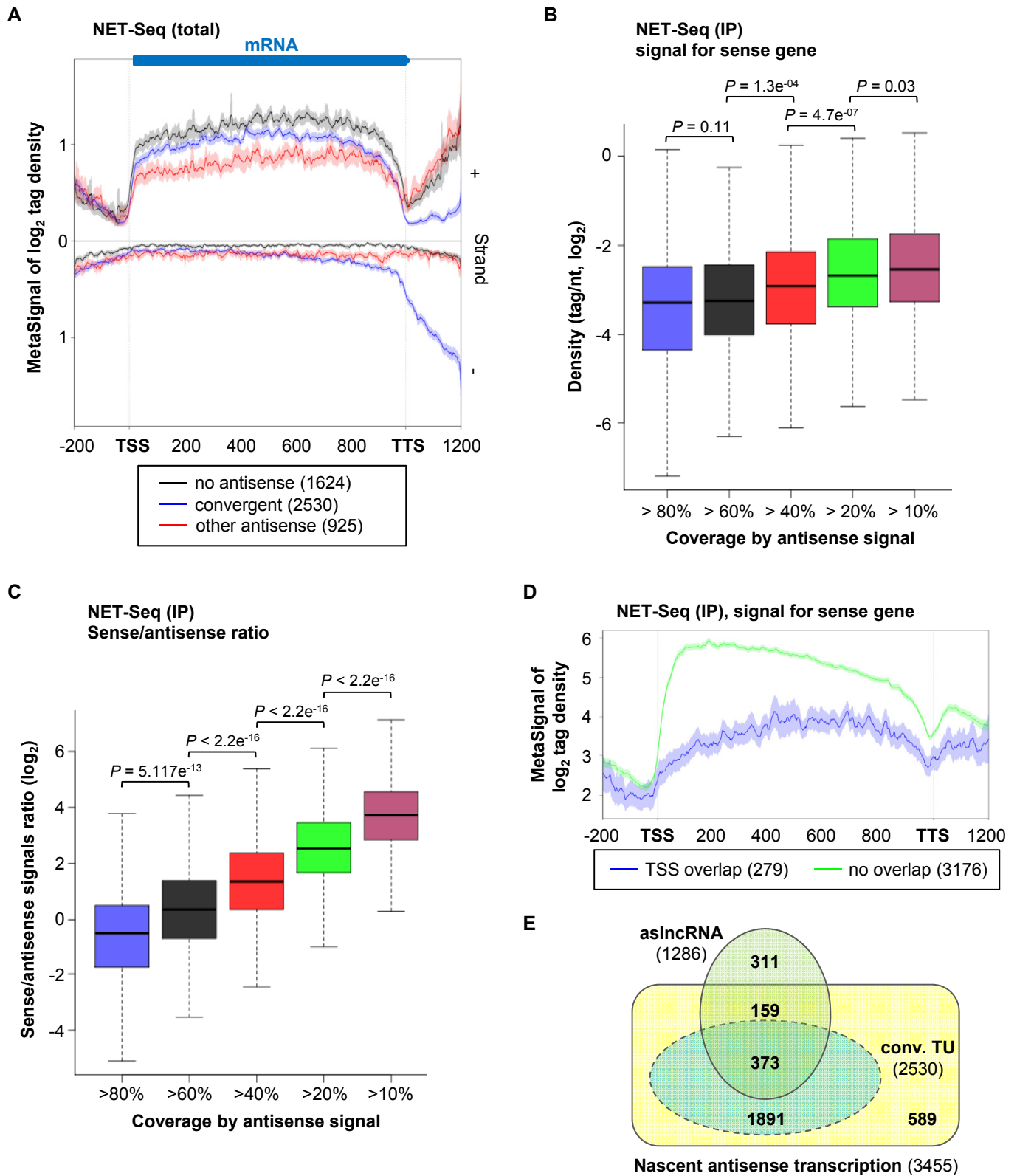

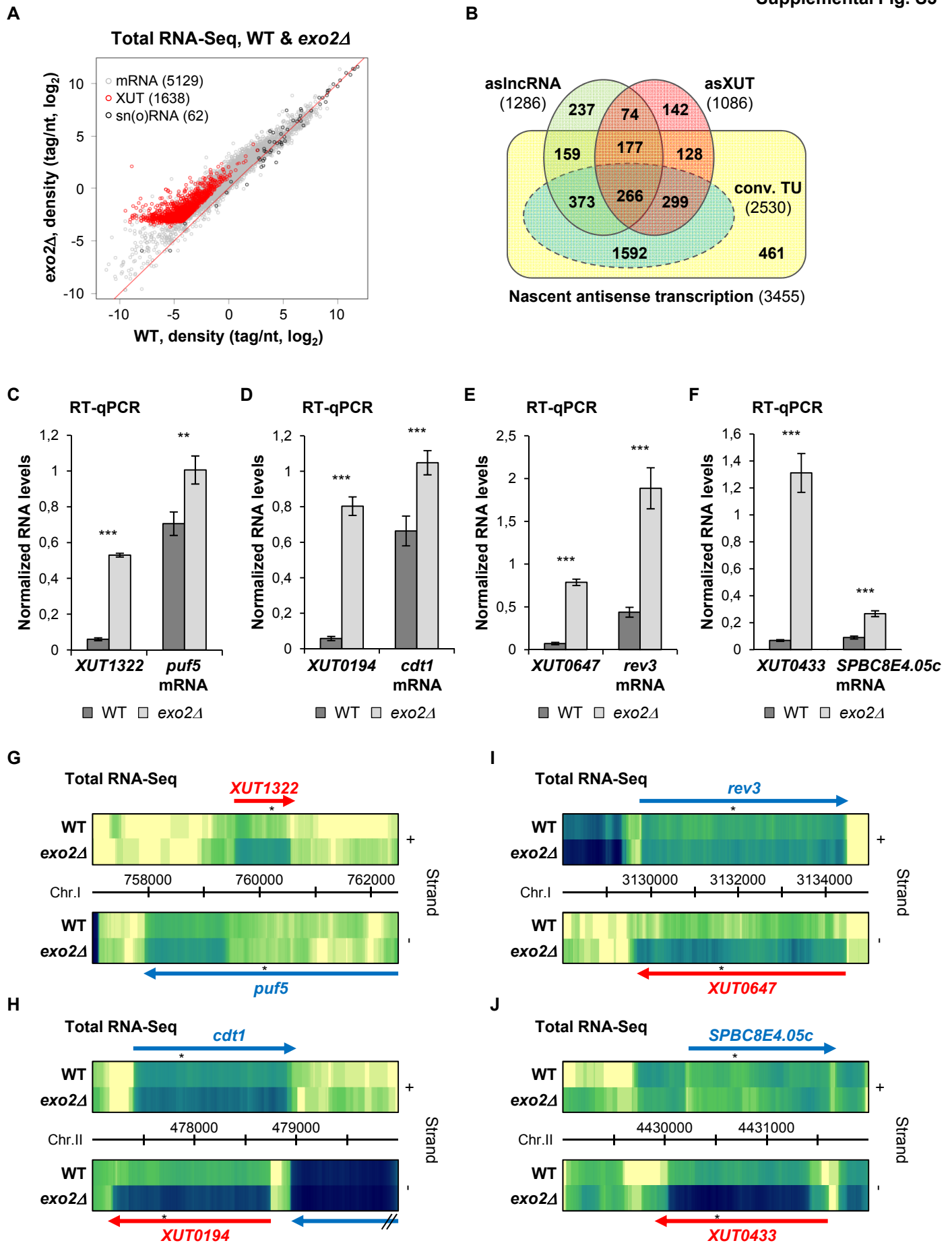

**A**

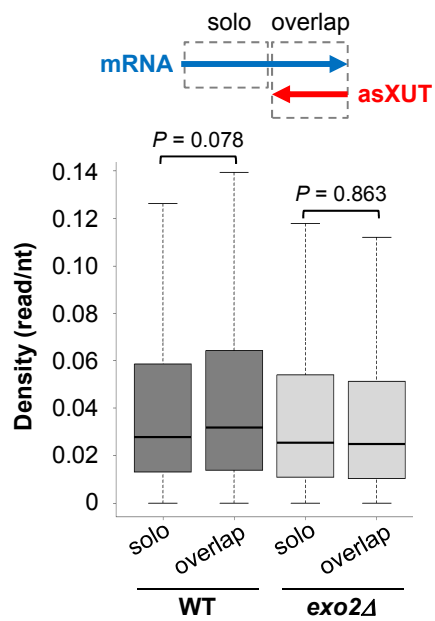

**B**

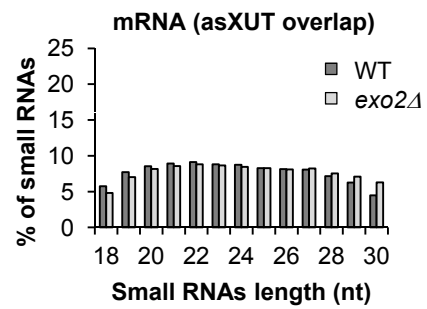

**C**

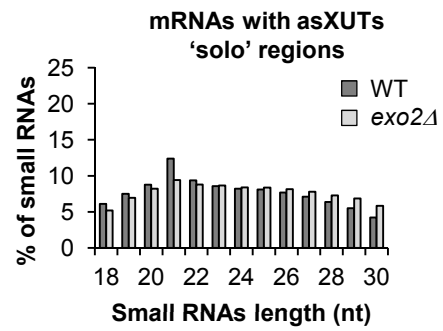

**D**

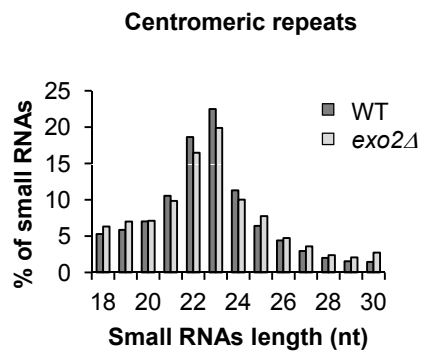

**E**

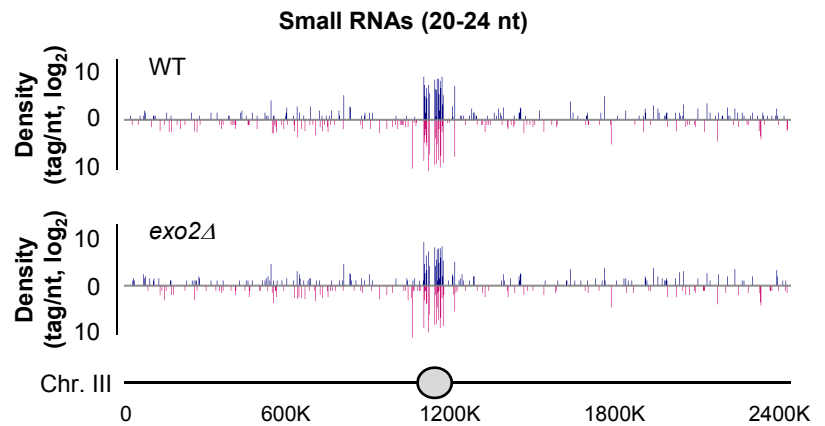

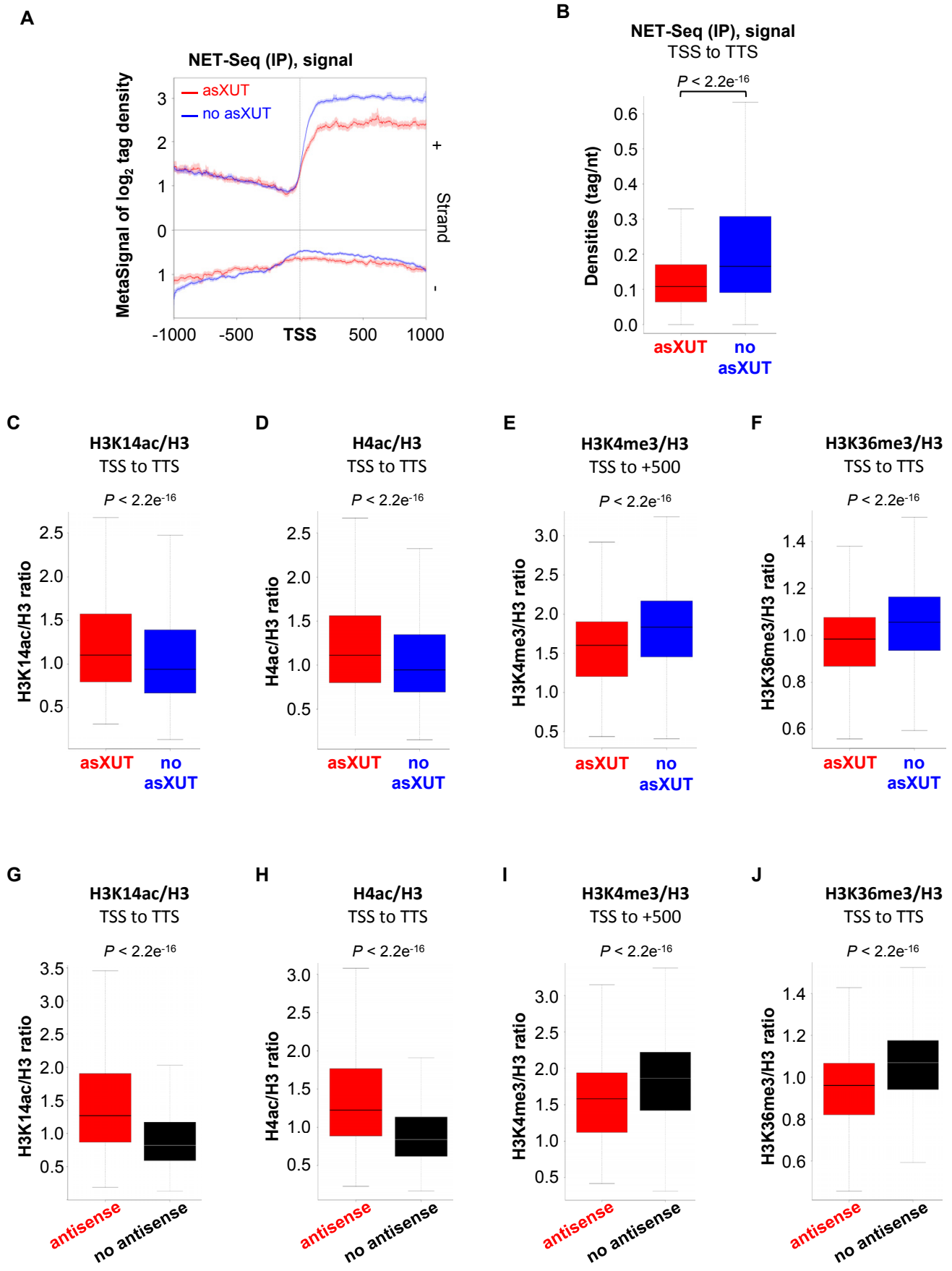

A

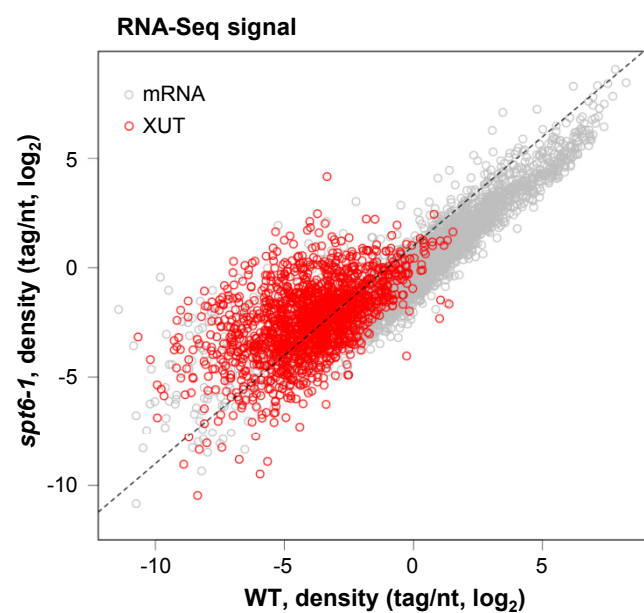

B

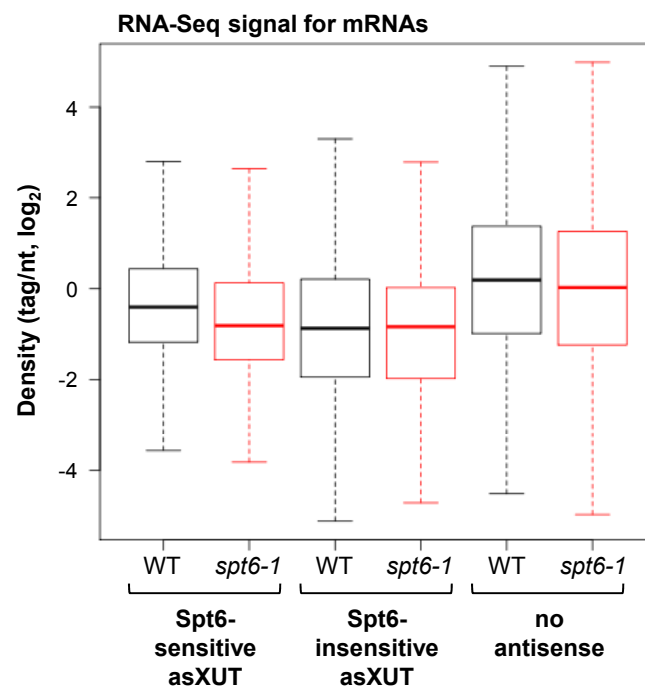

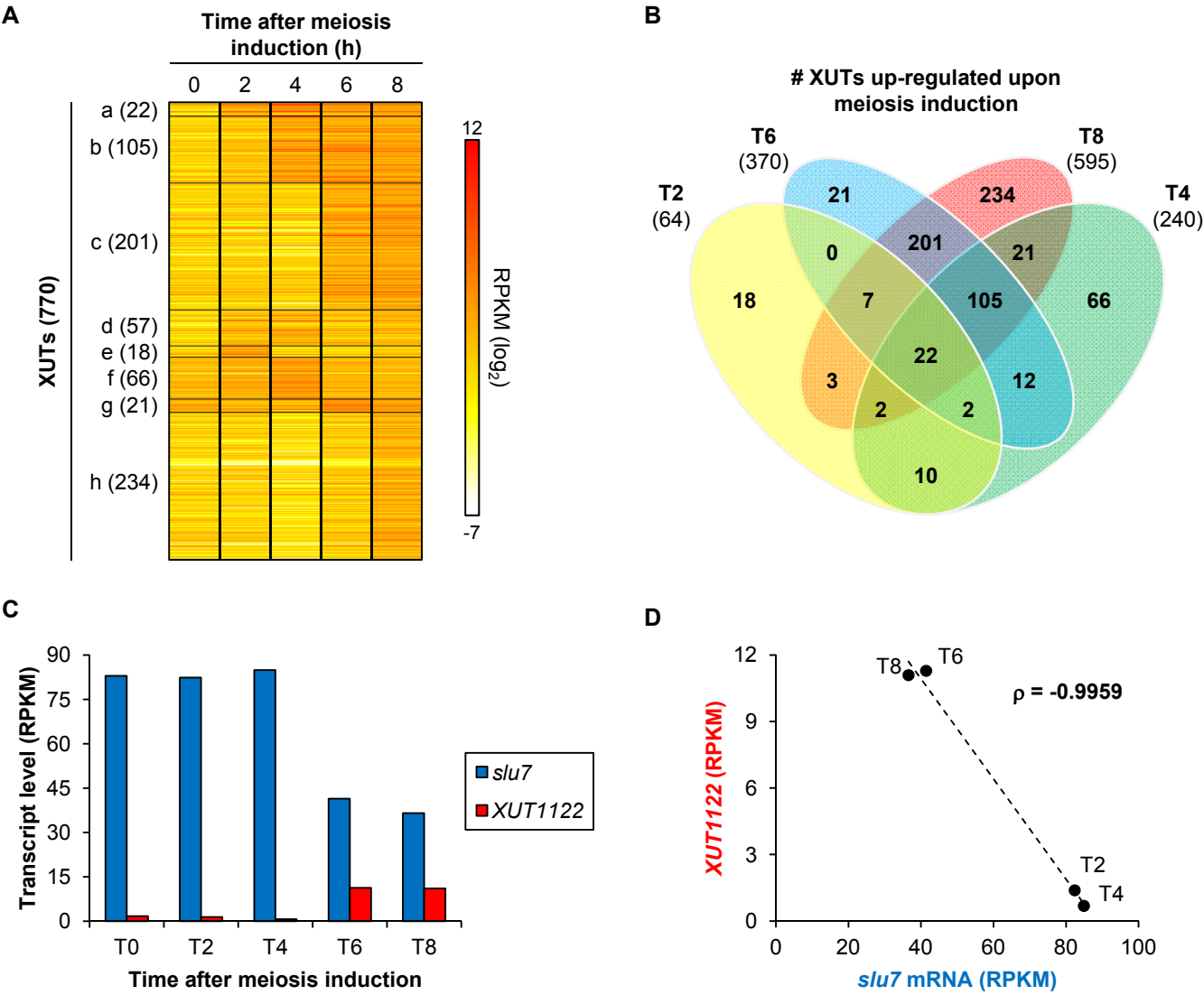

Supplement: Supplemental Material [file supp_063446.117_Supplemental_Figures.pdf]
